# Complementary transcriptome and proteome profiling in the mature seeds of *Camellia oleifera* from Hainan Island

**DOI:** 10.1371/journal.pone.0226888

**Published:** 2020-02-06

**Authors:** Zhouchen Ye, Yougen Wu, Zeeshan Ul Haq Muhammad, Wuping Yan, Jing Yu, Junfeng Zhang, Guanglong Yao, Xinwen Hu

**Affiliations:** Institute of Tropical Agriculture and Forestry, Hainan University, Haikou, Hainan Province, China; ICAR - National Research Center on Plant Biotechnology, INDIA

## Abstract

*Camellia oleifera* Abel. (*C*. *oleifera*), as an important woody tree species producing edible oils in China, has attracted enormous attention due to its abundant unsaturated fatty acids and their associated benefits to human health. To reveal novel insights into the characters during the maturation period of this plant as well as the molecular basis of fatty acid biosynthesis and degradation, we conducted a conjoint analysis of the transcriptome and proteome of *C*. *oleifera* seeds from Hainan Island. Using RNA sequencing (RNA-seq) technology and shotgun proteomic method, 59,391 transcripts and 40,500 unigenes were obtained by TIGR Gene Indices Clustering Tools (TGICL), while 1691 protein species were identified from Mass Spectrometry (MS). Subsequently, all genes and proteins were employed in euKaryotic Orthologous Groups (KOG) classification, Gene Ontology (GO) annotation, and Kyoto Encyclopedia of Genes and Genomes (KEGG) enrichment analysis to investigate their essential functions. The results indicated that the most abundant pathways were biological metabolic processes. There were 946 unigenes associated with lipid metabolism at the transcriptome level, with 116 proteins at the proteome level; among these, 38 specific proteins were involved in protein-protein interactions, with the majority being related to fatty acid catabolic process. The expression levels of 21 candidate unigenes encoding target proteins were further detected by quantitative real-time polymerase chain reaction (qRT-PCR). Finally, Gas Chromatography Mass Spectrometry (GC-MS) was carried out to determine the fatty acid composition of *C*. *oleifera* oil. These findings not only deepened our understanding about the molecular mechanisms of fatty acid metabolism but also offered new evidence concerning the roles of relevant proteins in oil-bearing crops. Furthermore, the lipid-associated proteins recognized in this research might be helpful in providing a reference for the synthetic regulation of *C*. *oleifera* oil quality by genetic engineering techniques, thus resulting in potential application in agriculture.

## Introduction

*Camellia oleifera* Abel. (*C*. *oleifera*), belonging to genus *Camellia* of the Theaceae family, is one of the world's four main woody oil tree species (together with the oil palm, olive, and coconut) [1–3]. It is a signature oil crop in China, with abundant resources and an annual output of over 2.4 million tons of seeds, and its oil yield is more than 0.6 million tons [4–7]. The crop covers a huge area of approximately 4.4 million hectares and is distributed in the Jiangxi, Hunan, Fujian, Hainan and Guangxi provinces [8–11]. Recently, *C*. *oleifera* has drawn increased attention because of its consumption by humans, its use in cosmetics production, and its ornamental value [12,13]. *C*. *oleifera* seeds are the raw materials for extracting the edible vegetable oil, which is highly admired due to its beneficial unsaturated fatty acids [14]. Thus, *C*. *oleifera* oil is referred to as “eastern olive oil”, and it can also be used for medicinal purposes according to the dictionary records of Chinese Materia Medica [15].

Due to its large-scale cultivation under different environmental conditions in China, *C*. *oleifera* populations have developed various morphological characteristics, growth habits, and oil qualities [16]. Hainan Island is the southernmost edge of the *C*. *oleifera* distribution, as the climate is unique on account of segregationist isolation [17]. As an outcross-pollinated plant, the dual effects of environmental factors and hereditary properties have given rise to rich and distinctive *C*. *oleifera* cultivars with abundant phenotypic variation [18]. The oil of this crop is traditionally used by local residents to treat burns, scalds, stomach troubles, oral ulcers, and cardiovascular and cerebrovascular diseases [19,20].

Nevertheless, in previous studies of *C*. *oleifera* from Hainan Island, researchers have mainly focused on genetic diversity [21,22], pathological identification [23], pharmacological components [24], and biological activity [25,26]. Indeed, the critical genes and proteins contained in seeds could affect the quality of *C*. *oleifera* oil by regulating the pathways of fatty acid biosynthesis and degradation. Although there are several researches concerning the transcriptome of the mature seeds in this plant [27,28], few studies have aimed to comprehensively understand the molecular foundations of lipid metabolism in *C*. *oleifera* from Hainan Island. In view of this, a combined analysis of the transcriptomics and proteomics of *C*. *oleifera* seeds from Hainan Island was performed using RNA sequencing (RNA-seq) technique and shotgun qualitative approach, both of which are powerful methods with the advantages of intensive coverage and great sensitivity. Afterward, several representative genes encoding target proteins were selected, and their expression levels were detected by quantitative real-time polymerase chain reaction (qRT-PCR) at four different developmental stages in order to identify the key candidate proteins associated with lipid metabolism, as well as to reveal the corresponding molecular mechanisms during *C*. *oleifera* ripening. In order to further evaluate the quality of its oil, Gas Chromatography-Mass Spectrometry (GC-MS) analysis was carried out to determine fatty acid content.

The targets of this study were to recognize the key candidate proteins associated with fatty acid biosynthesis and degradation, and to preliminarily investigate the potential genetic and molecular mechanisms of the corresponding metabolic pathways. Furthermore, the obtained datasets can not only be exploited for gene expression, protein profiling, and function annotation, but also expand our understanding of the characters of *C*. *oleifera* from Hainan Island in the fruit maturation period, thereby providing a theoretical reference for the utilization of *C*. *oleifera* germplasm resources and promoting its industrial development.

## Materials and methods

### Plant materials

Fresh *C*. *oleifera* fruits at full maturity stage that were uniform in size, shape, and color, and that had not been attacked by insects, were collected from Yangjiang town (19°12′10"N; 110°24′32"E), Qionghai city, Hainan province in China, on November 24^th^, 2018. This study was carried out on private land, and the owner of the land gave permission to conduct the research on this site. No specific permissions were required for this location, and the field studies did not involve endangered or protected species. To maximize the sample representativeness, approximately 2.0 kg of ripe and plump seeds accounting for 36.89% of whole fruits were obtained from 12 individual 10-year-old plants. In brief, each four plants represented a biological replicate containing about 0.7 kg of seeds, nearly 0.17 g seeds from each plant. These trees were selected as the research objects with the same good growth state, an average single plant fruit yield of 25 kg, an average height of 2.7 m, an average crown diameter of 3.3 m, and an average stem diameter of 10.6 cm. The distance between trees was at least 30 m based on the covered area of the *C*. *oleifera* experimental forest farm. Some seeds were wrapped in tin foil and then rapidly frozen under liquid nitrogen, and others were packed in sealed package bags. All samples were transferred to the laboratory for further analysis. Crucially, the same samples were prepared for extraction of RNA, protein, and oil.

For qRT-PCR, seeds of *C*. *oleifera* were harvested during the 2018 season at four different developmental periods: the nutrition synthesis stage (S1, August 24th), fat accumulation stage (S2, September 24th), near mature stage (S3, October 24th), and full maturity stage (S4, November 24th). The site, plants, and method of sampling are given above. The botanical characters of *C*. *oleifera* samples in various growth phases are summarized in S1 Table.

### Transcriptomic analysis

#### RNA extraction

Mature seeds of *C*. *oleifera* were homogenized under liquid nitrogen, and total RNA was extracted using an RNA-prep Pure Plant Kit (TIANGEN, China) according to the manufacturer's instructions. The concentration and quality of RNA were determined using an Agilent 2100 Bioanalyzer with an Agilent RNA 6000 Nano Kit (Agilent Technologies, USA). The RNA Integrity Number (RIN) was 8 or greater. Furthermore, the purity of RNA was measured using a NanoDrop^TM^ Ultraviolet Spectrophotometer (Thermo Fisher Scientific, USA). Three biological replicates were performed for all samples to maximize the reliability of statistical analysis.

#### Complementary DNA library construction and transcriptome sequencing

The cDNA library construction and transcriptome sequencing of *C*. *oleifera* seeds were carried out by OE Biotech. Co., Ltd. (Shanghai, China). The raw RNA-seq data were deposited in the NCBI Sequence Read Archive (SRA, http://www.ncbi.nlm.nih.gov/Traces/sra/) under accession numbers SRR10428065, SRR10428066 and SRR10428067, respectively. In brief, mRNA was enriched by oligo (dT) magnetic beads, then fragmentation buffer was added to fragment the mRNA into short pieces. Using mRNA as a template, the first strand cDNA was synthesized by random hexamer-primers. Thereafter, buffer, dNTPs, DNA polymerase I and RNase H were added to synthesize the second strand cDNA. The double-stranded cDNA was purified with a QiaQuick PCR Purification Kit (QIAGEN, Germany) and eluted with EB buffer. The end-repair was subjected followed by addition of Poly (A) and ligation of adapters. The agarose gel electrophoresis was used to select fragment size. Finally, the acquired fragments were enriched by PCR amplification, and the completed cDNA library was sequenced on an Illumina HiSeq X Ten platform (Illumina Inc., San Diego, CA, USA) [29].

#### Transcriptome assembly, annotation of genes, and functional classification

Raw sequence reads were processed to remove the low-quality reads containing adapters. In addition, the reads with a content of unknown bases N greater than 5% were also filtered out. The de novo transcriptome assembly was performed on the clean reads by Trinity software (v.2.2.0) with default parameters [30,31]. Then, the assembled transcripts were clustered using TIGR Gene Indices Clustering Tools (TGICL) to remove redundancy, and the expressed genes were ultimately obtained.

The program BLASTx was used to assign putative functions to all genes based on the following databases: NCBI Non-Redundant (NR) protein database, Swiss-Prot protein database, euKaryotic Orthologous Groups of proteins (KOG) database, and Kyoto Encyclopedia of Genes and Genomes (KEGG) database. Moreover, according to the NR annotation information, BLAST2GO (http://www.blast2go.com/) analysis was carried out to offer automatic Gene Ontology (GO) annotation for every gene, and then the assembled genes were classified into three GO categories. The protein sequences were predicted using TransDecoder software to establish a protein database of *C*. *oleifera* seeds for subsequent proteomic analysis (**S1 File**).

### Proteomic analysis

#### Protein preparation and quantification

Total proteins were extracted with the Borax/PVPP/Phenol (BPP) method from mixed seeds of *C*. *oleifera* as described previously with some modifications [32]. Briefly, samples were rapidly frozen in liquid nitrogen before grinding with mortar and pestle. During this process, a small amount of polyvinylpolypyrrolidone (PVPP) was added to minimize proteolysis and protein degradation. Approximately 3.0 g of freeze-dried powder was added into three volumes of extraction buffer, and then the mixture solution was vortexed thoroughly for 10 min at room temperature and centrifuged at 15,000 g for 15 min at 4°C. The upper phase was transferred to a new centrifuge tube. This process was repeated two more times, and then the extract solutions were combined, and mixed with isovolumetric Tris-saturated phenol (pH > 7.8). Similarly, the mixing solution was vortexed and centrifuged under the same conditions. The supernatant was cautiously transferred to another new centrifuge tube containing isochoric extraction buffer and mixed vigorously on a vortex mixer, and the final phenol-based upper phase was collected by centrifugation for 15 min. This extraction step was repeated once more to remove the impurities from the mixtures. Subsequently, proteins were precipitated by mixing with 5 ml saturated ammonium sulfate methanol solution and incubating at -20°C overnight.

The extracted proteins were gathered through centrifugation at 12,000 rpm for 30 min at 4°C and rinsed two times with 100% cold methanol and acetone. Then, the resulting proteins were air-dried for 3–5 min at room temperature and dissolved in about 500 μl lysis buffer for more than 2 h at 22°C. After centrifugation for 30 min as described above, the protein concentration was measured according to the Bradford assay method using a UV-160 Spectrophotometer (Shimadzu, Japan) with Bovine Serum Albumin (BSA) as the standard. These proteins immediately underwent shotgun analysis or were stored at -80°C.

#### Shotgun proteomic analysis of *C*. *oleifera* seeds

About 300 μg of proteins from *C*. *oleifera* mature seeds were reduced and alkylated using Dithiothreitol (DTT) and Iodoacetamide (IAM), followed by digestion with trypsin for 16 h at 37°C. The peptide segments of enzymatic hydrolysis were separated via an UltiMate 3000 system equipped with a Pepmap C_18_ column (Thermo Fisher Scientific, USA) following a 65 min 5–35% organic gradient. Thirty washed fractions were collected at one-minute intervals and further freeze-dried, then subjected to a High-resolution Mass Spectrometer Triple TOF 6600 system (SCIEX, USA). These peptides were automatically selected, and the corresponding proteins were identified through the ProGroup^TM^ algorithm and ProteinPilot^TM^ software V5.0 (SCIEX, USA) to calculate the error factor (EF), reporter peak area and p value as described by Xuchu Wang et al. [33]. A protein database was established using target protein sequences predicted via TransDecoder software based on the transcriptome data. Proteins with an unused score > 1.3 (confidence ≥ 95%) were considered as positively identified. Finally, an in-house BlastP search was conducted for all proteins to recognize their homologues and potential functions. Three biological replicates of proteins from *C*. *oleifera* seeds were normalized to the intensity of qualitatively matched proteins (or peptides).

#### Protein function classification, hierarchical clustering, pathway and STRING analysis

The functions of identified proteins were confirmed by searching against the databases. First, the Venn diagram was constructed via the Draw Venn Diagram website (http://bioinformatics.psb.ugent.be/webtools/Venn/). Following this, the subcellular localization was predicted through CELLO V.2.5 (http://cello.life.nctu.edu.tw/), which generated reductions according to a two-level support vector machine system [34]. The proteins were then assigned to the KOG database (http://www.ncbi.nlm.nih.gov/COG/). GO annotation and KEGG pathway analysis of recognized proteins were performed using GENE DENOVO Omicshare Tools Cloud Platform software (http://www.omicshare.com/tools/Home/Index/index.html). Moreover, in order to further determine their protein-protein interaction networks, the STRING V.9.1 database (http://www.string-db.org/cgi/input.pl) with a high confidence view (score 0.9) was utilized.

### qRT-PCR assay

Total RNA from mixed seeds of *C*. *oleifera* was isolated using an RNA-prep Pure Plant Kit (TIANGEN, China) according to the manufacturer’s recommendations [35]. The concentration and quality of RNA were determined using an Agilent 2100 Bioanalyzer with an Agilent RNA 6000 Nano Kit (Agilent Technologies, USA). A RIN of 8 or greater was required. Furthermore, the purity of RNA was measured using a NanoDrop^TM^ Ultraviolet Spectrophotometer (Thermo Fisher Scientific, USA). First-strand cDNA was further synthesized by a RevertAid First Strand cDNA Synthesis Kit (Thermo Fisher Scientific, USA). To normalize the expression data, the Actin gene (glyceraldehyde-3-phosphate dehydrogenase, GAPDH) was used as an internal reference, and primer pairs for amplification of the selected genes were designed through Primer 5.0 software (Premier Biosoft, USA) [36,37]. The qRT-PCR assay was carried out with ChamQ^TM^ Universal SYBR^®^Qpcr Master Mix (Vazyme Biotech, China) on an Applied Biosystems StepOnePlus^TM^ (Thermo Fisher Scientific, USA), using the following program: 95°C for 30 s, followed by 40 cycles of 95°C for 10 s, and then 60°C for 30 s. The relative expression levels of target genes were calculated with the 2^-ΔΔCt^ method [38]. All qRT-PCR experiments included three independent biological replicates for each sample. Gene-specific primer sequences and detailed information are given in S9 Table.

### GC-MS analysis of fatty acids

#### Standard preparation

A mixture of 37 fatty acid methyl ester standards from C6 to C24 with certificates of composition was purchased from Nu Chek Prep Inc. (Elysian, MN, USA) and diluted with n-hexane to standard solutions at different concentration gradients, then passed through 0.45 μm syringe filters prior to analysis.

#### *C*. *oleifera* seed pretreatment

**Oil extraction.** The seeds of *C*. *oleifera* were air-dried and ground into powder (60 mesh), then stored in a drying oven for further analysis. In total, about 300 g of mixed seed powder was weighed and soaked in petroleum ether using a SOX 406 Soxhlet Extractor (Hanon Instruments, China) for oil extraction. The refined petroleum ether was then reused for extraction. The oil was stored at ambient temperature for further treatment. Three independent biological experiments were conducted.

**Sample hydrolysis.** A moderate amount of *C*. *oleifera* oil was mixed with about 100 mg of pyrogallic acid in a round-bottom flask, and then 2.0 ml of 95% ethanol solution and 10 ml of hydrochloric acid solution were added, and the mixtures were shaken vigorously and incubated in a water bath at 70°C–80°C for 40 min. After hydrolysis, the flask was cooled to room temperature.

**Fat extraction.** The hydrolyzed sample was mixed with 10 ml 95% ethanol solution and vortexed fully. Subsequently, the hydrolysate was transferred from the flask to a separation funnel containing 50 ml of petroleum ether and shaken for 5 min and then allowed to stand for 10 min. Finally, the ether layer extract was collected into a 250 ml flask. The flask was steamed in a water bath and dried for 2 h in an oven at 100°C ± 5°C.

**Fat saponification and methyl esterification.** The sample needed to be treated with fat saponification and methyl esterification before the detection of fatty acids in *C*. *oleifera* oil, based on the MIDI protocol [39] and GB 5009.168–2016. All solutions needed to be filtered through 0.45 μm membrane filters before use.

#### GC-MS determination

The GC-MS analysis was performed with three replicates of *C*. *oleifera* samples using an Agilent 7890B-7000B GC-MS (Agilent Technologies, USA) instrument equipped with an HP-5MS silica capillary column (0.25 mm × 30 m × 0.25 μm). The injector and interface were maintained at 250°C. The thermal program was as follows: heating from 60 to 270°C at 6°C per minute and holding at 270°C for 2 min. The splitless injection mode was used, and the injected volume was 1.0 μl. Helium with a purity of 99.995% was used as the carrier gas at a flow rate of 1.0 ml/min. The split ratio was 10:1.

The MS conditions were as follows: ion source, electron ionization (EI); ion source temperature, 230°C; EI mode, 70 eV; mass range, 50–450 mz^−1^; electron multiplier voltage, 2.0 kV; and full-scan mode with a solvent delay of 4 min. The components were identified based on their unique retention indices (RIs) in accordance to the National Institute of Standards and Technology (NIST) database. Then, the absolute content of fatty acids were calculated based on the corresponding regression equations [40].

#### Statistical analyses

The data were reported as mean ± standard deviation (n = 3). All results were statistically analyzed by one-way analysis of variance (ANOVA) in SPSS 19.0 software package for Windows (SPSS Inc., Chicago, IL, USA). Pearson’s correlation coefficient analysis was carried out, with correlations at p < 0.05 considered significant. All determinations were done at least in triplicate.

## Results

### Transcriptome sequencing and de novo assembly

**Fig 1:**
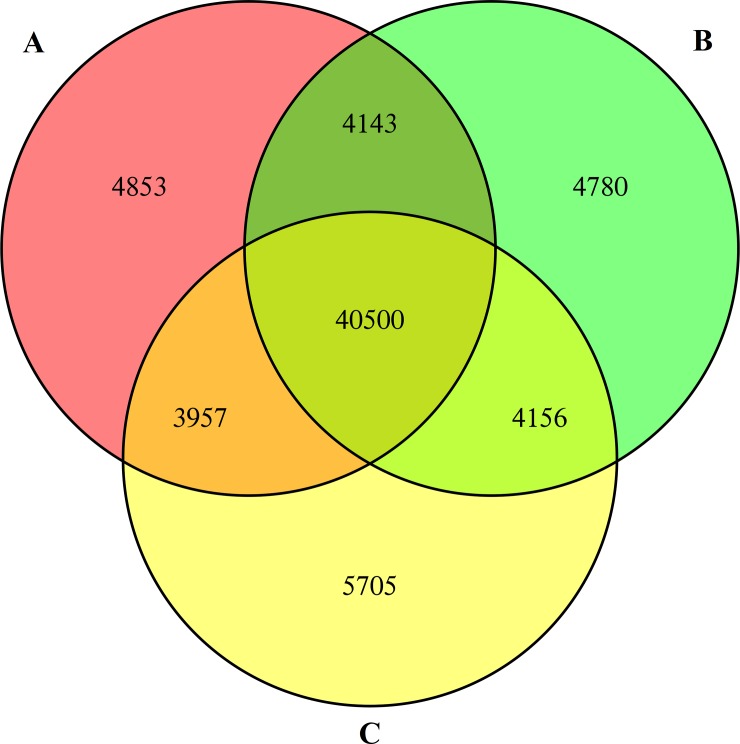
**Venn diagram of the identified unigenes from *C*. *oleifera* seeds by RNA-Seq in three biological duplicates (A, B and C).** Numbers in the overlapping regions refer to those unigenes that were expressed in more than one sample. In total, 68,094 unigenes were identified at least once, 52,756 unigenes were acquired from at least two tests, and 40,500 shared unigenes were obtained in all three experiments. The detailed identities of these expressed unigenes are listed in S2 Table.

In the present study, a total of 54,131,736 reads were generated. After removing duplicate reads and trimming adaptors and low-quality sequences, 52,422,046 paired-end 100 bp clean reads of *C*. *oleifera* seeds were obtained. The reads, with a GC content of 47.39% and Q30 percentage of 94.53%, were selected for assembly using Illumina HiSeq X Ten sequencing technology. Among the three biological replications, the reads were assembled into 59,391 transcripts. From these, 40,500 unigenes were further identified via matching gene sequences in databases, where the lengths ranged from 201 bp to 15,804 bp. The average length of these unigenes was 713 bp, and their N50 value was 1025 bp (Fig 1 and Table 1).

**Table 1:** Data assembly for Trinity in the digital transcriptome of *C*. *oleifera* seeds.

Type	Amount	GC%	N50	Min Length	Mean Length	Max Length	Assembled Bases
Genes	40500	41.65	1025	201	713	15804	28870625
Transcripts	59391	41.51	962	201	708	15804	42075258

### Function annotation and classification

**Fig 2:**
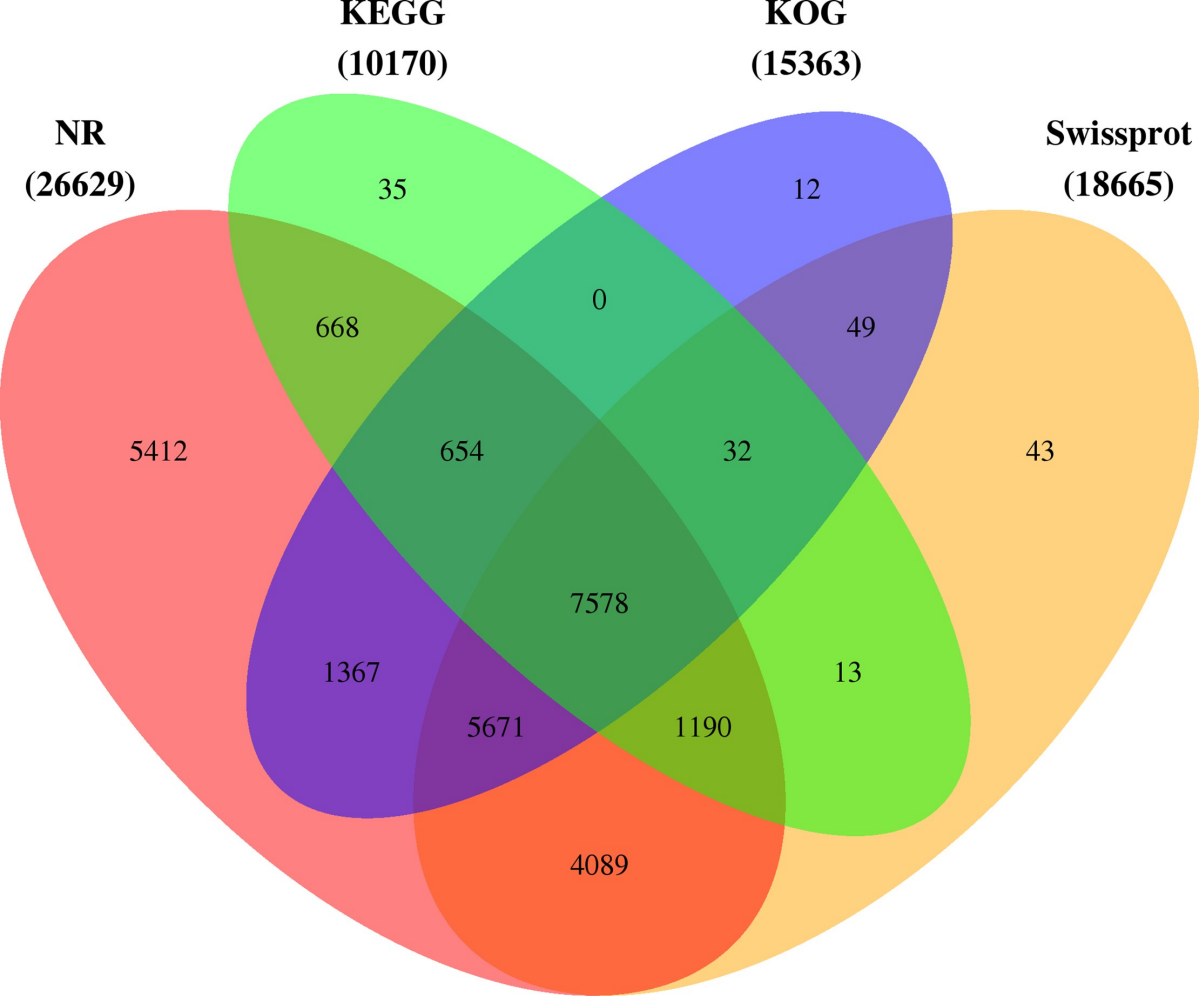
The KEGG, KOG, Nr, and Swiss-Prot database annotations of Venn diagram of expressed unigenes. The co-expressed unigenes from *C*. *oleifera* seeds in three biological repeats were annotated and incorporated by the KEGG, KOG, Nr, and Swiss-Prot databases. In total, 26,813 unigenes matched with at least one database, and 7578 shared unigenes matched with all four databases.

The genes were searched against the specific databases using their protein-coding sequences to predict their functions. A total of 26,813 unigenes (approximately 66.20% of all unigenes) were annotated in NR, Swiss-Prot, KOG, GO and KEGG databases, as indicated in Fig 2.

**Fig 3:**
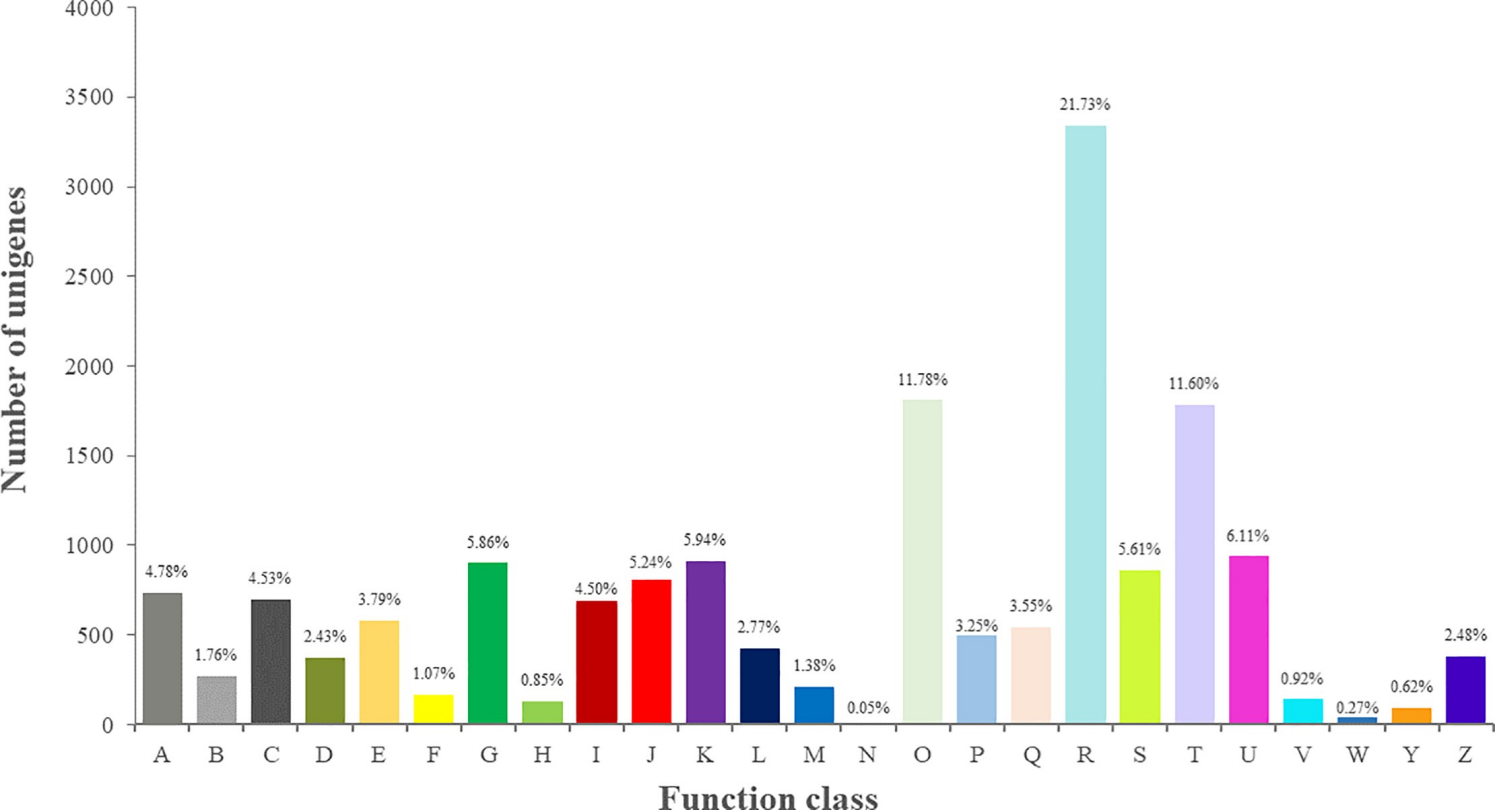
KOG functional categories of the expressed unigenes from *C*. *oleifera* seeds. Numbers and percentages of these unigenes in different clusters are summarized, and the abbreviations are as follows: A. RNA processing and modification; B. Chromatin structure and dynamics; C. Energy production and conversion; D. Cell cycle control, cell division, chromosome partitioning; E. Amino acid transport and metabolism; F. Nucleotide transport and metabolism; G. Carbohydrate transport and metabolism; H. Coenzyme transport and metabolism; I. Lipid transport and metabolism; J. Translation, ribosomal structure and biogenesis; K. Transcription; L. Replication, recombination and repair; M. Cell wall/membrane/envelope biogenesis; N. Cell motility; O. Posttranslational modification, protein turnover, chaperones; P. Inorganic ion transport and metabolism; Q. Carbohydrate transport and metabolism; R. General function prediction only; S. Function unknown; T. Signal transduction mechanisms; U. Intracellular trafficking, secretion, and vesicular transport; V. Defense mechanisms; W. Extracellular structures; Y. Nuclear structure; Z. Cytoskeleton.

All genes were searched against the KOG database to divide ortholog clusters by phylogenetical relations. A total of 15,363 unigenes (37.93%) were classified into 25 functional clusters (Fig 3 and S2 Table). The five largest categories were (1) general function prediction only (3339, 21.73%), (2) posttranslational modification, protein turnover, chaperones (1809, 11.78%), (3) signal transduction mechanisms (1782, 11.60%), (4) intracellular trafficking, secretion, and vesicular transport (938, 6.11%), and (5) transcription (912, 5.94%).

**Fig 4:**
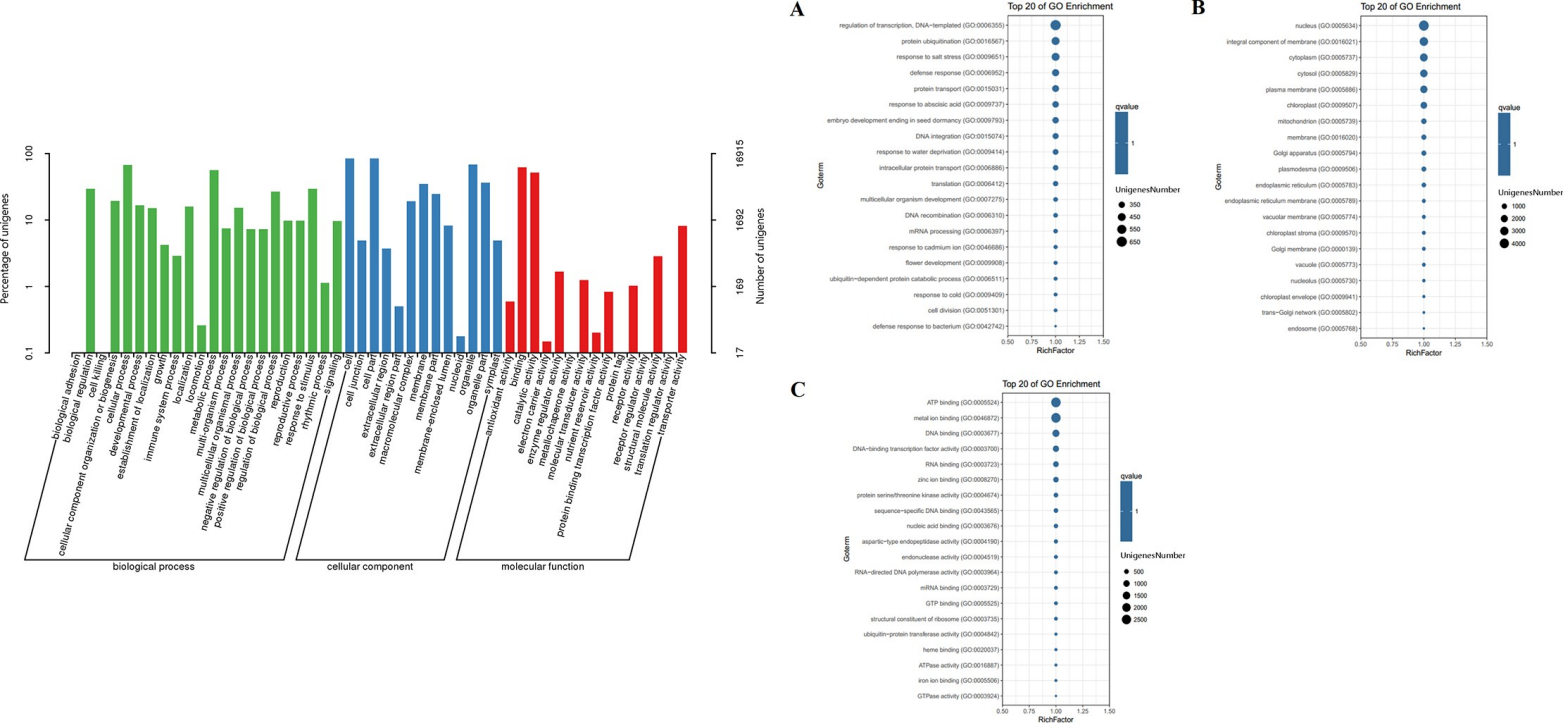
GO classification of the expressed unigenes from *C*. *oleifera* seeds. The unigenes were assigned to three main categories: biological process (A), cellular component (B), and molecular function (C). The sizes of circular rings, ranging from 350 to 4000, represent the numbers of unigenes involved in the most abundant 20 terms of each category.

Furthermore, the genes with homologues in the NR database were annotated to GO classifications. In total, the identified unigenes were assigned GO pathways and categorized into 50 terms in three categories that consisted of biological processes, molecular functions, and cellular components (Fig 4 and S3 Table). With regard to biological processes (Fig 4A), “Cellular process” (11,507 unigenes) was the largest subcategory, followed by “Metabolic process” (9528 unigenes), “Response to stimulus” (5013 unigenes), and “Biological regulation” (5006 unigenes). Within the cellular component categories (Fig 4B), the top three clades were “Cell” (14,292 unigenes), “Cell part” (14,265 unigenes), “Organelle” (11,621 unigenes), and “Organelle part” (6181 unigenes). Among the molecular functions (Fig 4C), the major GO terms were “Binding” (10,495 unigenes), “Catalytic activity” (8788 unigenes), and “Transporter activity” (1377 unigenes).

**Fig 5:**
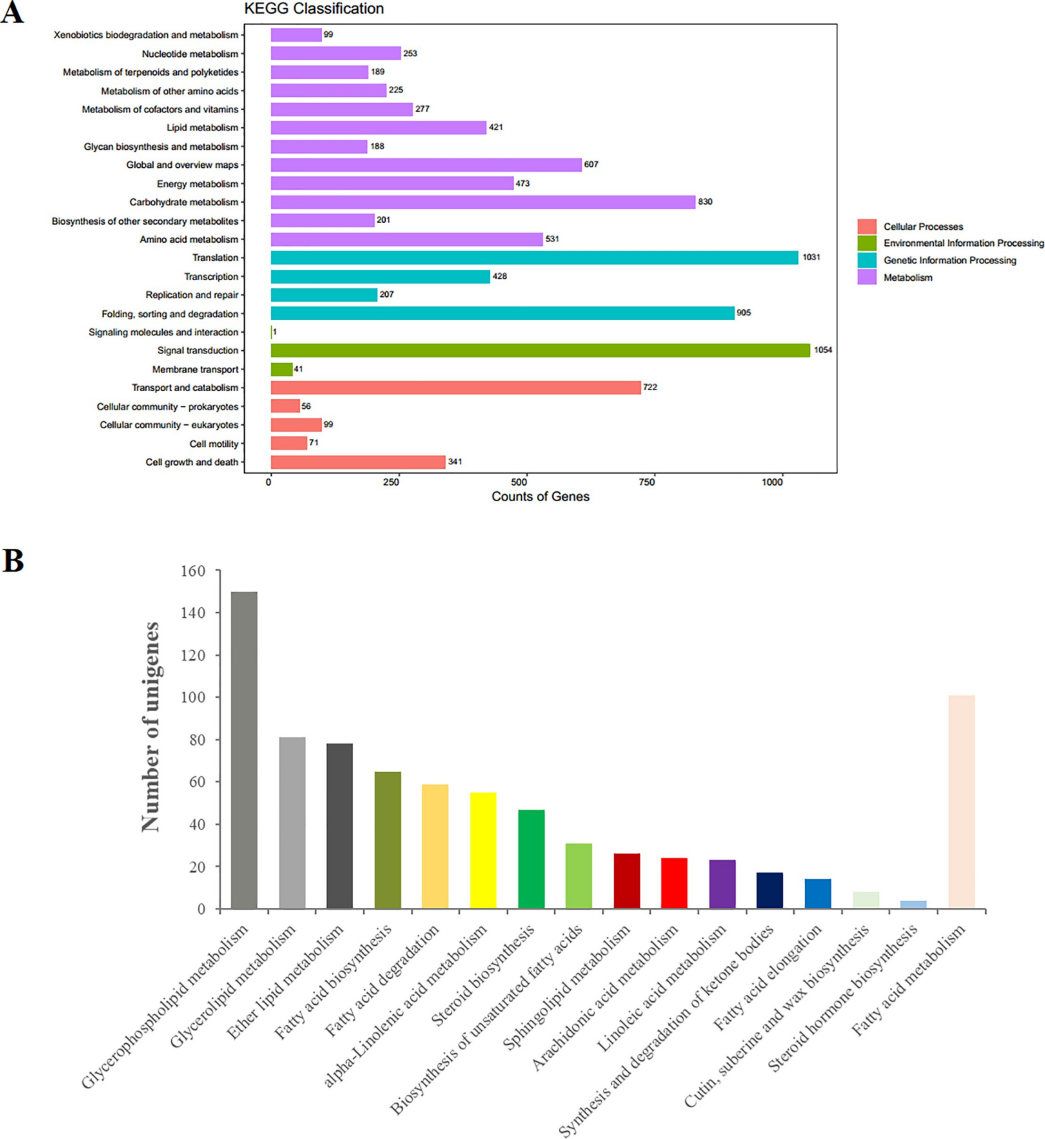
**KEGG pathway analysis of the expressed unigenes from *C*. *oleifera* seeds (A).** The unigenes were categorized into four main portions: metabolism, genetic information processing, environmental information processing, and cellular processes. The numbers of unigenes enriched in the KEGG database are marked on the right side. **Sub-pathways of the special unigenes, which related to lipid metabolism (B).** The 522 unigenes involved in lipid metabolism pathway were divided into 16 subclasses.

Finally, a total of 10,170 unigenes (25.11%) were annotated to 24 biological pathways in the KEGG database (Fig 5A and S4 Table). These pathways were subdivided into four categories. “Signal transduction” represented the most abundant group, followed by “Translation” and “Folding, sorting and degradation”. Moreover, the pathways associated with “Carbohydrate metabolism”, “Transport and catabolism”, “Amino acid metabolism”, and “Energy metabolism” were also highly represented. It was worth noting that the lipid metabolism pathway containing 16 subclasses of 522 unigenes was extremely critical (Fig 5B and S4 Table). Among these, 150 unigenes were involved in glycerophospholipid metabolism, 101 in fatty acid metabolism (S1 Fig), and 81 in glycerolipid metabolism, followed by 78 unigenes, 65 unigenes, 59 unigenes, 55 unigenes, 31 unigenes, 23 unigenes, and 14 unigenes that were associated with ether lipid metabolism, fatty acid biosynthesis (S2 Fig), fatty acid degradation (S3 Fig), alpha-linolenic acid metabolism (S4 Fig), biosynthesis of unsaturated fatty acids (S5 Fig), linoleic acid metabolism (S6 Fig), and fatty acid elongation (S7 Fig), respectively.

**Fig 6:**
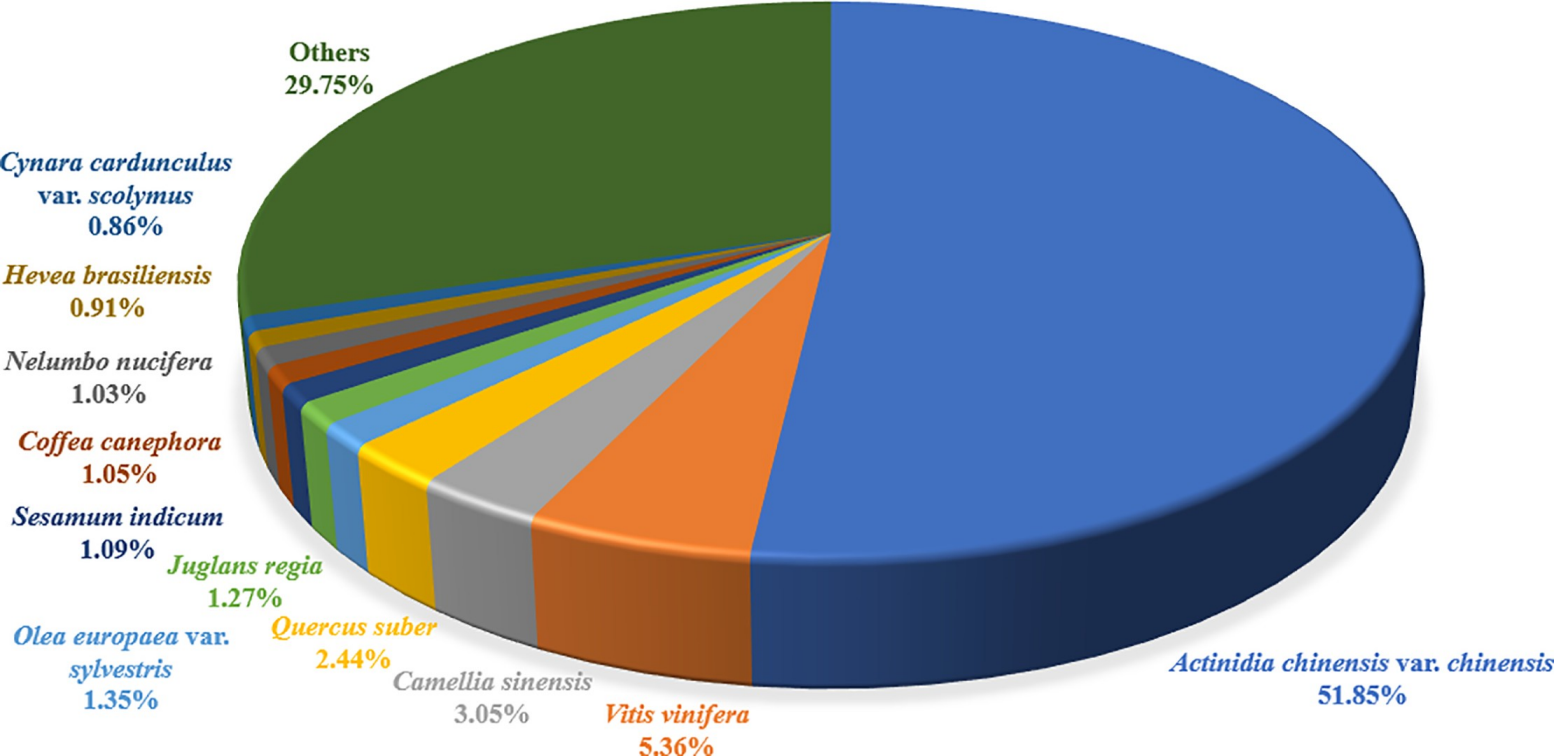
Species distribution for the expressed unigenes of *C*. *oleifera* seeds. These unigenes had high sequence homologies to 299 different species, and 11 major groups are presented.

Against the NR database, the distributions of species in different plants were obtained (Fig 6 and S2 Table), revealing that the first group of 13,800 unigenes had high sequence homology to *Actinidia chinensis* var. *chinensis*, followed by 1426 unigenes to *Vitis vinifera*, 812 unigenes to *Camellia sinensis*, and 650 unigenes to *Quercus suber*. In addition, a relatively large group included 359 unigenes matched to genes from *Olea europaea* var. *sylvestris*, 338 unigenes to *Juglans regia*, and 289 unigenes to *Sesamum indicum*.

### Identification of proteins from *C*. *oleifera* seeds by high-throughput shotgun proteomic analysis

**Fig 7:**
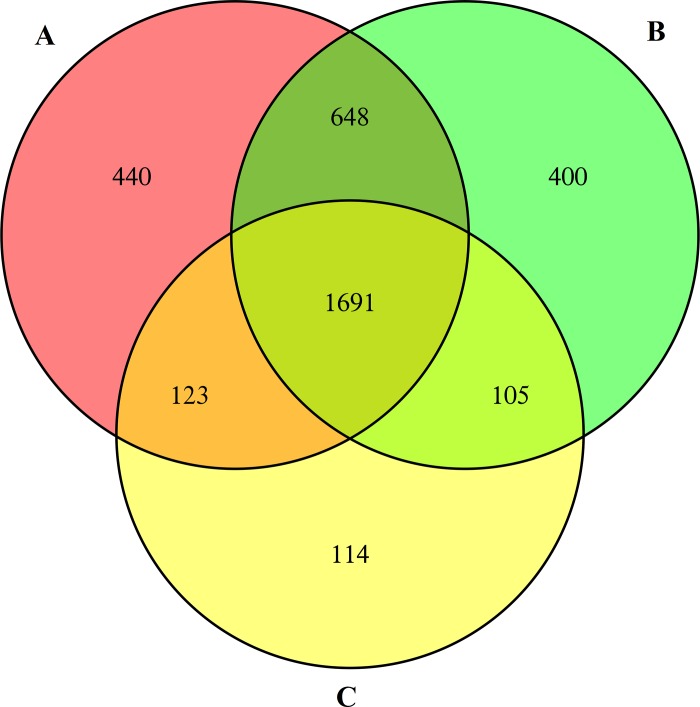
**Venn diagram of the identified proteins from *C*. *oleifera* seeds by shotgun in three biological duplicates (A, B, and C).** The overlapping regions indicate the numbers of common proteins. A total of 3521 proteins were expressed at least once; 2567 proteins were acquired from at least two tests, and 1691 shared proteins were obtained in all three experiments. The detailed identities of these proteins are listed in S5 Table.

The high-throughput shotgun is a second-generation proteomic technique using gel-free methods for protein identification. Lower abundance proteins that are difficult to isolate by 2-DE can be identified via shotgun. The extracted proteins obtained from *C*. *oleifera* seeds were exposed to digestion to attain the peptides for LC-MS/MS analysis, followed by a database search with Protein Pilot (Cutoff Applied: >0.05 (10%), Protein Pilot^™^ Software 5.0, Triple TOF AB 5600). Finally, we identified 2602, 2844, and 2033 proteins from three independent shotgun experiments (Unused (Conf) Cutoff >1.3 (95%)). In total, 3521 proteins were examined at least once; among these, 2567 proteins were identified from two replications, while 1691 shared proteins were detected in all three experiments (Fig 7). In the following study, the positively identified proteins from three repetitions were nominated to be the finally recognized proteins. The main objective of this research was to analyze the critical metabolic pathways in which these identified proteins were involved and to describe their important regulatory functions.

### Functional classification and annotation

**Fig 8:**
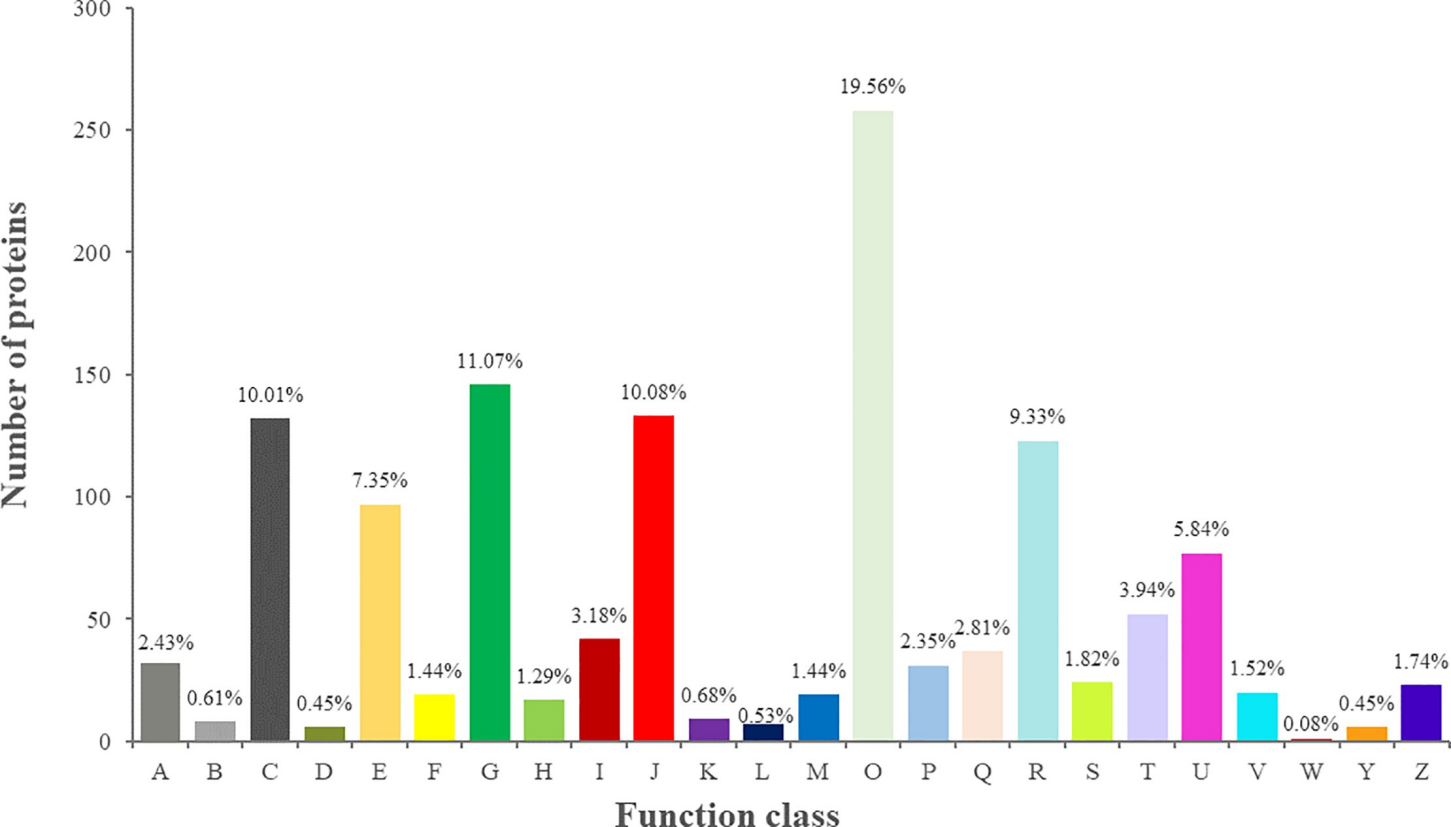
KOG functional categories of the identified proteins from *C*. *oleifera* seeds. Numbers and percentages of the proteins in different clusters are presented, and the abbreviations are as follows: A. RNA processing and modification; B. Chromatin structure and dynamics; C. Energy production and conversion; D. Cell cycle control, cell division, chromosome partitioning; E. Amino acid transport and metabolism; F. Nucleotide transport and metabolism; G. Carbohydrate transport and metabolism; H. Coenzyme transport and metabolism; I. Lipid transport and metabolism; J. Translation, ribosomal structure and biogenesis; K. Transcription; L. Replication, recombination and repair; M. Cell wall/membrane/envelope biogenesis; O. Posttranslational modification, protein turnover, chaperones; P. Inorganic ion transport and metabolism; Q. Carbohydrate transport and metabolism; R. General function prediction only; S. Function unknown; T. Signal transduction mechanisms; U. Intracellular trafficking, secretion, and vesicular transport; V. Defense mechanisms; W. Extracellular structures; Y. Nuclear structure; Z. Cytoskeleton.

The functional categories of all annotated proteins were deduced from sequence similarity with identified ortholog species. First, each of the identified proteins was functionally classified by KOG analysis (Fig 8 and S5 Table). The proportion of each category was presented as the sum of the proportion of all identities. According to KOG functional classification, 1691 specific proteins were divided into 24 categories on the basis of their primary biological functions; 19.56% of the proteins were engaged in posttranslational modification, protein turnover, chaperones; 11.07% of the proteins were involved in carbohydrate transport and metabolism; 10.08% of the proteins were related to translation, ribosomal structure and biogenesis; 10.01% of the proteins were involved in energy production and conversion; 7.35% of the proteins participated in amino acid transport and metabolism; 5.84% of the proteins were associated with intracellular trafficking, secretion, and vesicular transport; 3.18% of the proteins took part in lipid transport and metabolism.

**Fig 9:**
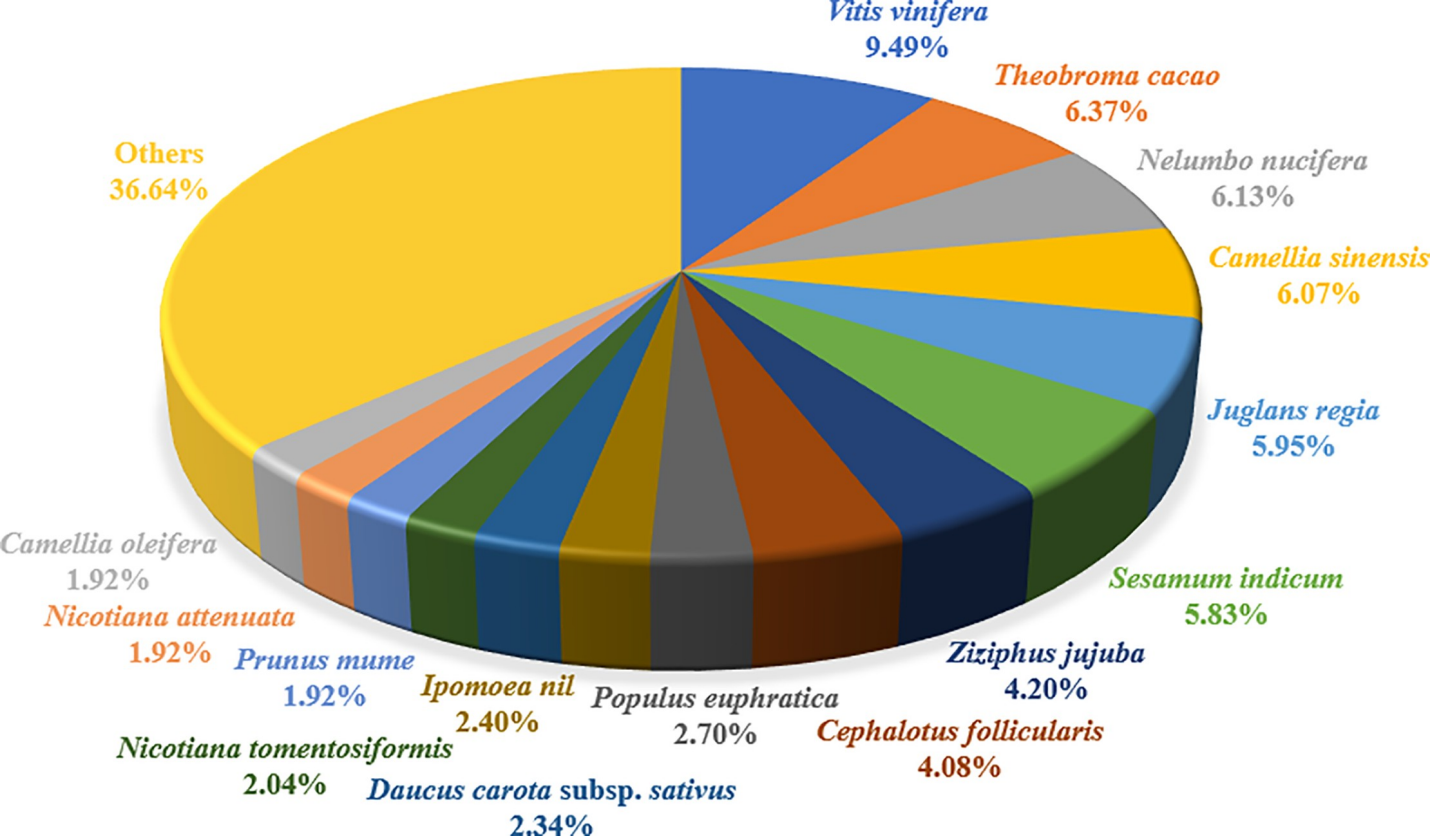
Species distribution for the identified proteins of *C*. *oleifera* seeds. These proteins had high sequence homologies to 126 different species, and 15 major groups are presented.

**Fig 10:**
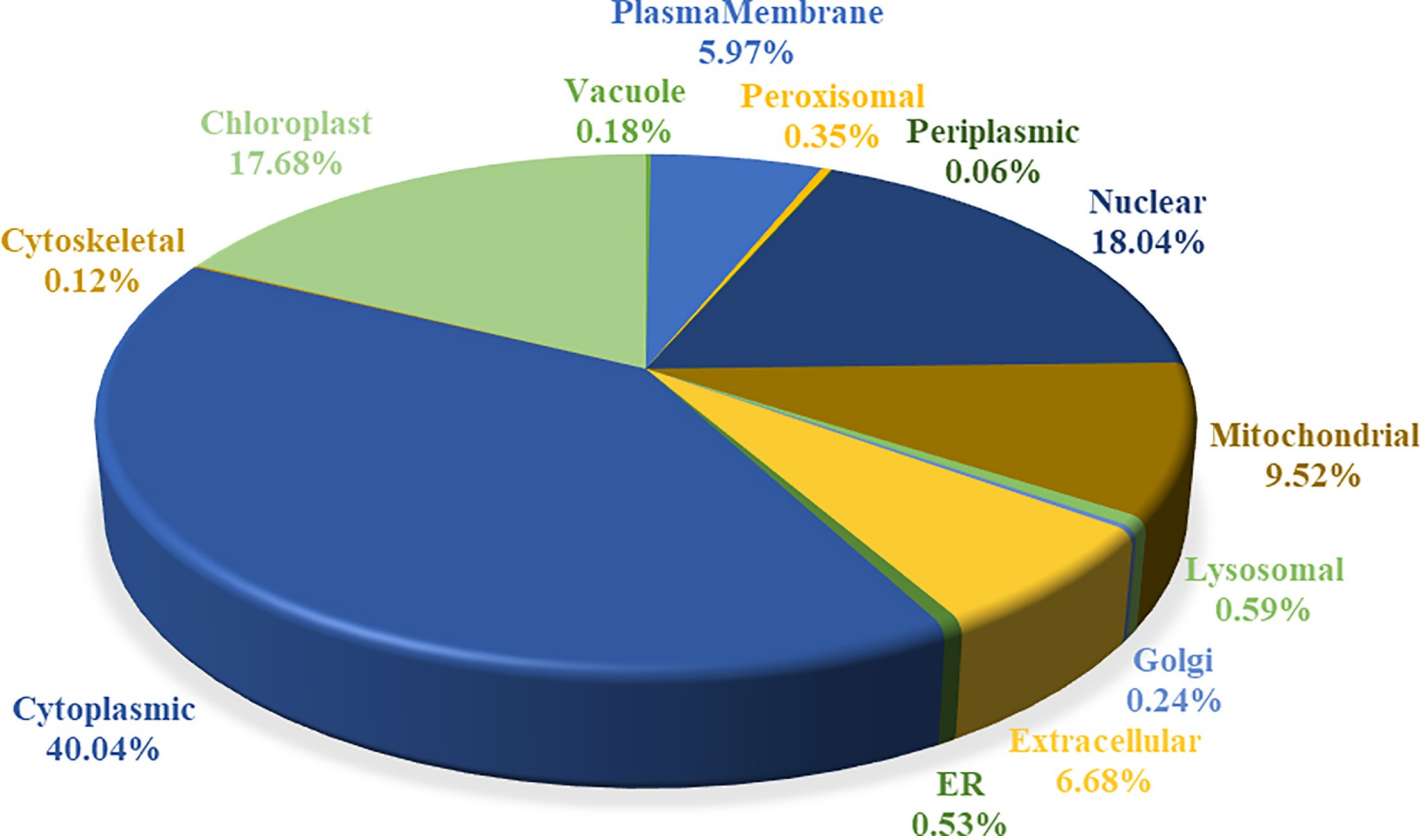
Subcellular location for the identified proteins of *C*. *oleifera* seeds. The subcellular locations of the proteins were classified into 13 main categories, and the proportion of each category is presented as the sum of the proportion of all identities.

As shown in Fig 9 and S5 Table, among the identified proteins, the largest proportion including 158 proteins had high sequence homology to *Vitis vinifera*, followed by 106 proteins to *Theobroma cacao*, 102 proteins to *Nelumbo nucifera*, and 101 proteins to *Camellia sinensis*. In addition, subcellular locations of these proteins were further predicted (Fig 10 and S5 Table), the first category containing 40.04% of cytoplasm-located proteins (677), and the second group composing of 299 proteins located in chloroplasts. Several proteins were also located in the extracellular space, plasma membrane, mitochondria, lysosome, peroxisome, vacuoles, and Golgi apparatus. These results indicated that a large number of proteins were associated with carbohydrate metabolism, biogenesis and energy production, essentially situated in the cytoplasm and chloroplast.

### Pathway analysis and protein-protein interaction of all identified proteins

**Fig 11:**
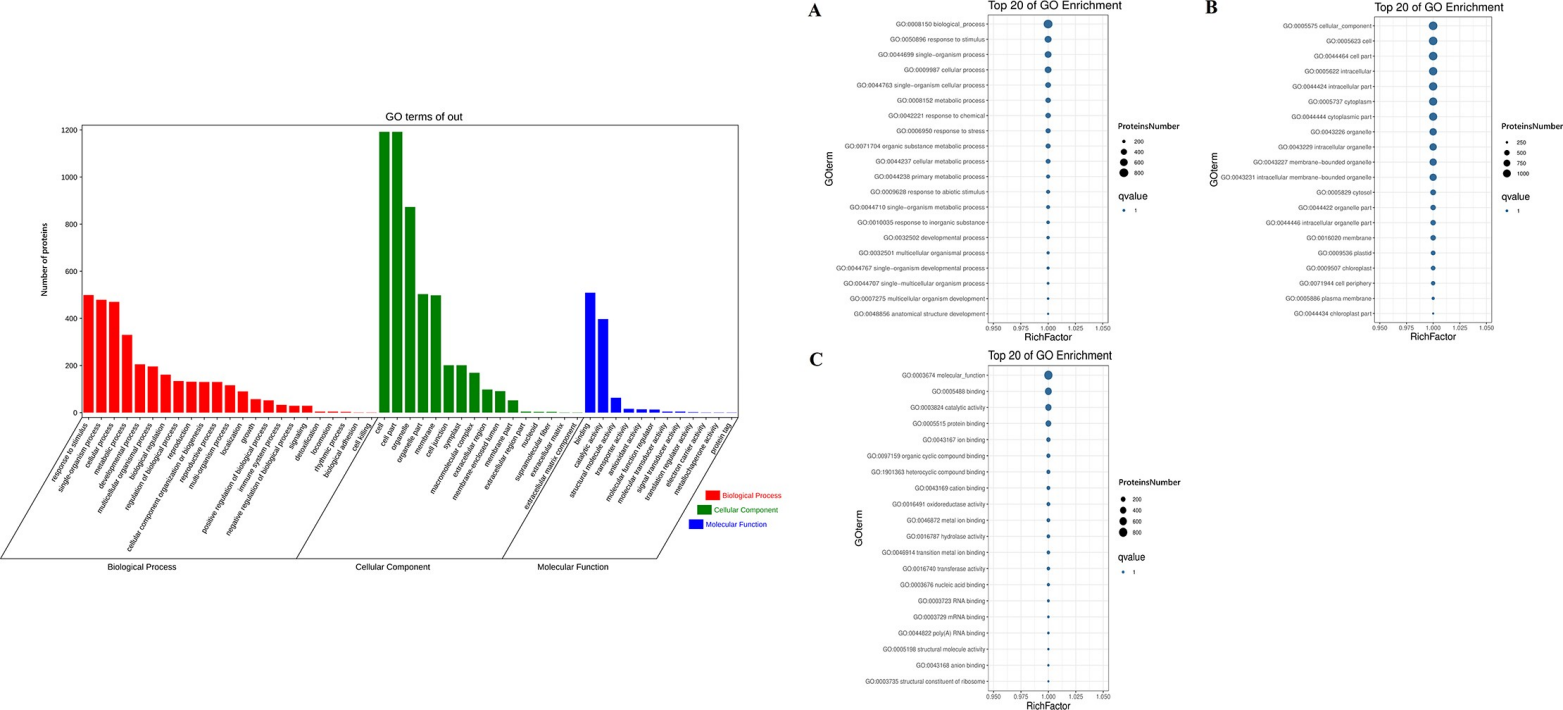
GO classification of the identified proteins from *C*. *oleifera* seeds. The proteins were assigned to three main categories: biological process (A), cellular component (B), and molecular function (C). The sizes of the circular rings, ranging from 200 to 1000, represent the numbers of proteins involved in the most abundant 20 terms of each category.

To further investigate the biochemical functions of recognized proteins from *C*. *oleifera* seeds, we performed GO analysis using the GENE DENOVO Omicshare Tools Cloud Platform software and assigned proteins to the categories of biological process, cellular component and molecular function (Fig 11 and S6 Table). The 1407 identified proteins were divided into three large groups comprising 51 subclasses based on their functional annotations. From the biological process perspective (Fig 11A), 23 classes of 814 proteins were determined. Within the classes, the largest group contained 499 proteins related to response to stimulus (GO:0050896); the second group contained 479 proteins involved in single-organism process (GO:0044699); the third group contained 470 proteins associated with cellular process (GO:0009987), followed by organic substance metabolic process (GO:0071704), cellular metabolic process (GO:0044237), and primary metabolic process (GO:0044238). There were also many proteins relevant to some other significant biological processes, for example, developmental process (GO:0032502), multicellular organismal process (GO:0032501), biological regulation (GO:0065007), regulation of biological process (GO:0050789), reproduction (GO:0000003), and reproductive process (GO:0022414). At the cellular component level (Fig 11B), 16 groups of 1219 proteins were observed. Among these, the larger subcategories were localized to intracellular part (GO:0044424), cytoplasmic part (GO:0044444), intracellular organelle (GO:0043229) and membrane-bounded organelle (GO:0043227). Furthermore, other proteins or isoforms occurred in the cell periphery (GO:0071944), plasma membrane (GO:0005886), cell-cell junction (GO:0005911) and endomembrane system (GO:0012505). Regarding molecular function ontology (Fig 11C), 12 pathways of 809 proteins were assigned. Almost half of these proteins participated in binding pathway; 326 proteins engaged in protein binding (GO:0005515), 157 in ion binding (GO:0043167), 135 in organic cyclic compound binding (GO:0097159), and 131 in heterocyclic compound binding (GO:1901363). This was followed by oxidoreductase activity (GO:0016491) and hydrolase activity (GO:0016787). It was worth noting that 98 proteins showed transferase activity (GO:0016740), participating in catalysis of the transfer from one compound to another. These regulatory pathways usually lead to posttranslational modifications. These different GO term distribution patterns in *C*. *oleifera* seeds might be relevant to their biological functions and might reveal some pivotal synthesis mechanisms of secondary metabolism.

**Fig 12:**
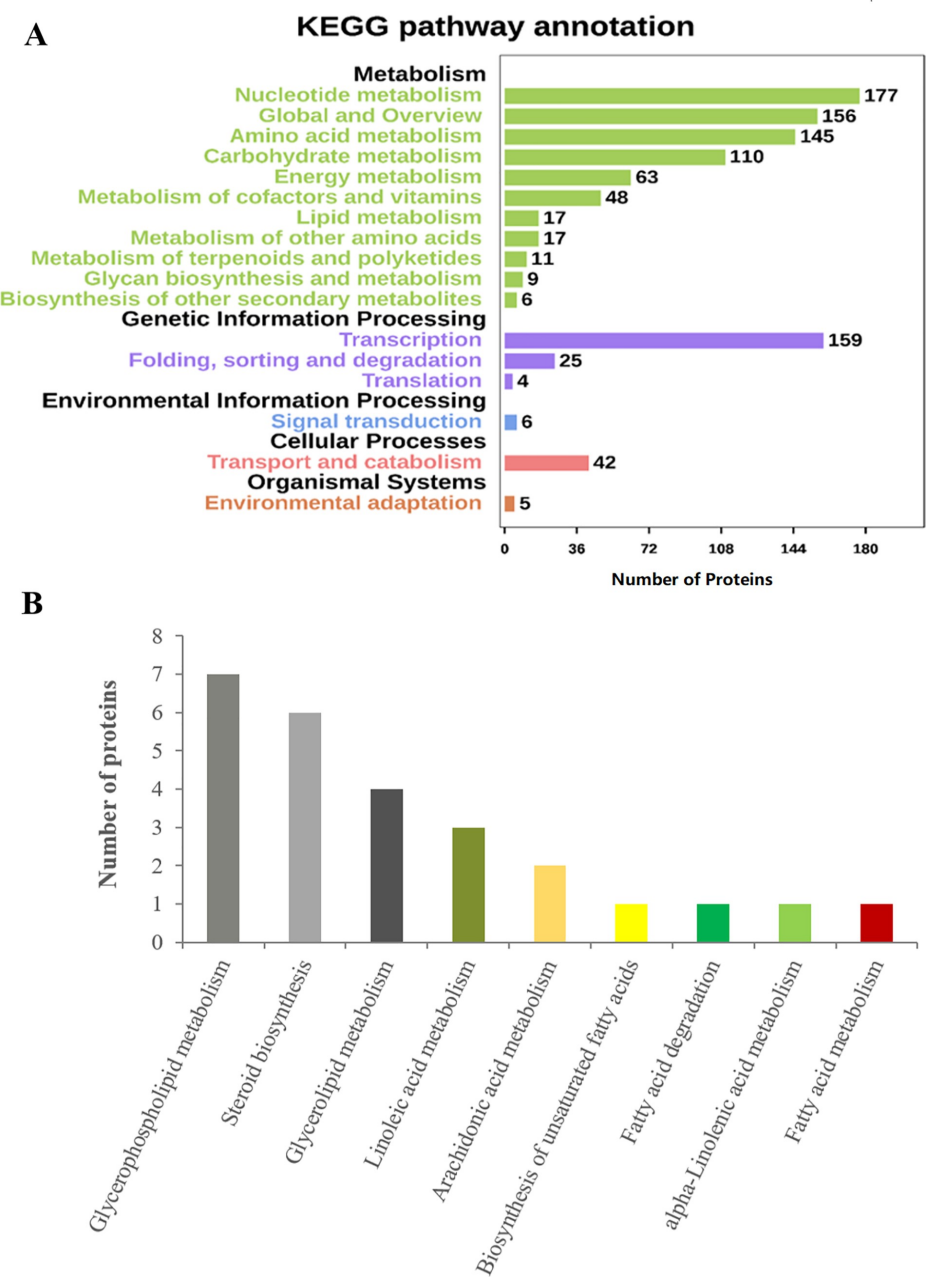
**KEGG pathway analysis of the identified proteins from *C*. *oleifera* seeds (A).** The proteins were categorized into five main portions: metabolism, genetic information processing, environmental information processing, cellular processes, and organismal systems. The numbers of proteins enriched in the KEGG database are marked on the right side. **Sub-pathways of the unique proteins, which were involved in lipid metabolism (B).** The 17 proteins involved in lipid metabolism pathway were divided into nine subclasses.

In order to obtain in-depth understanding of the biological processes these proteins were involved in, KEGG pathway analysis was performed. A total of 823 proteins were mapped to 17 KEGG pathways (Fig 12A and S7 Table). The most abundant proteins were annotated to the nucleotide metabolism pathway, followed by translation, amino acid metabolism, and carbohydrate metabolism pathways. Additionally, the lipid metabolism pathway of 17 proteins was particularly meaningful (Fig 12B and S7 Table); this category contained fatty acid metabolism (ko01212), biosynthesis of unsaturated fatty acids (ko01040), fatty acid degradation (ko00071), linoleic acid metabolism (ko00591), alpha-linolenic acid metabolism (ko00592), and arachidonic acid metabolism (ko00590) involved in the biosynthesis and degradation of fatty acids, indicating that these proteins with lipid metabolism might play crucial roles in the quality of *C*. *oleifera* oil.

**Fig 13:**
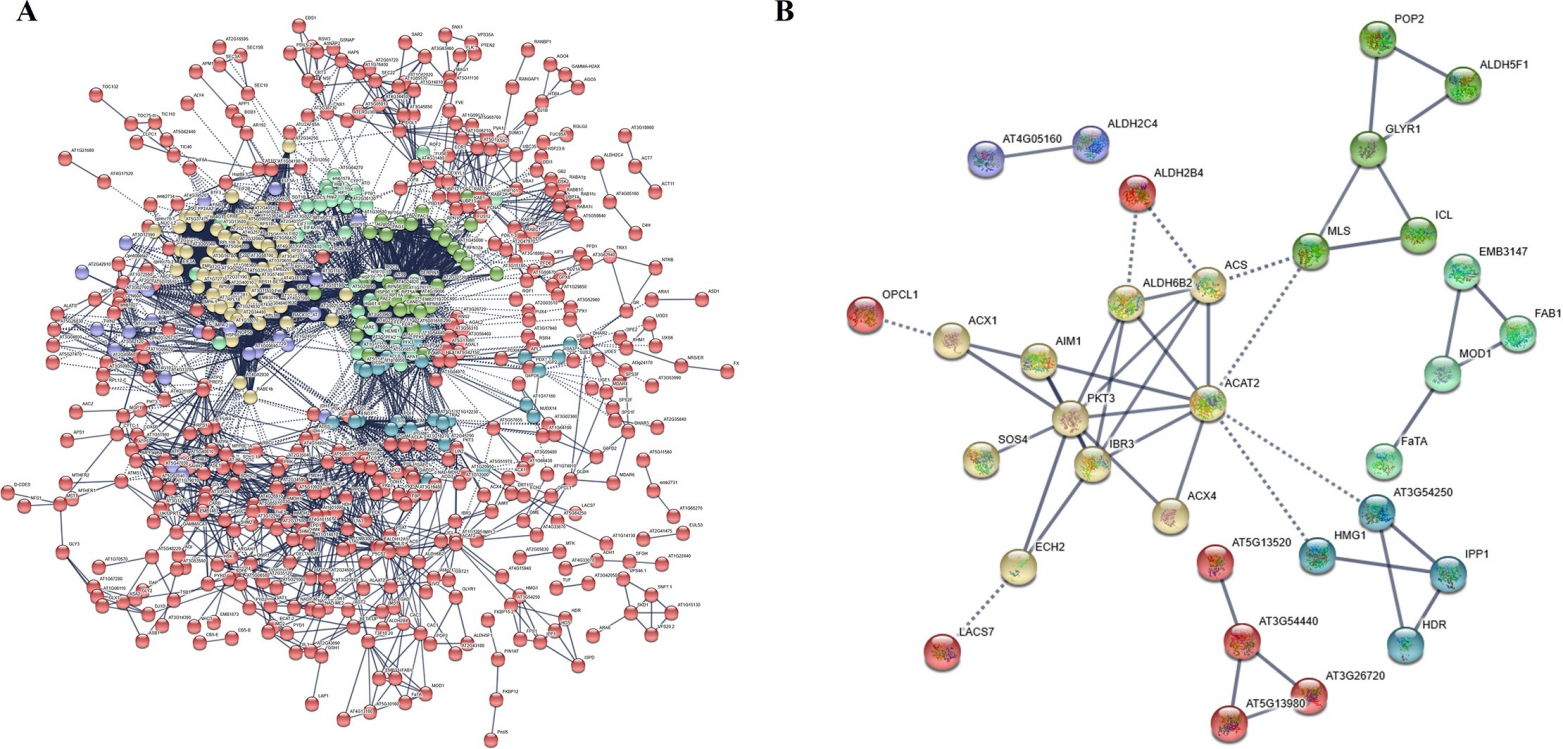
**Protein-protein interactions of the identified proteins from *C*. *oleifera* seeds (A).** A total of 660 proteins were involved in protein-protein interactions that comprised six main clusters. **Interactions among the unique proteins involved in lipid metabolism pathway (B).** The 38 interacted proteins associated with lipid metabolism were grouped into six subclusters.

In addition, the identified proteins were analyzed using the STRING database to determine their protein-protein interactions. This approach has been highlighted in several high throughput studies [41]; nevertheless, no data were available from the STRING database for *C*. *oleifera* or its sibling species. Therefore, these proteins first needed to be transformed into *Arabidopsis thaliana* proteins on the basis of their protein sequences, followed by selection of the *Arabidopsis thaliana* database for further analysis. In this study, a high confidence view (score 0.90) was used to determine the protein-protein interaction network. The network nodes and edges represented proteins and predicted functional correlations, respectively. Finally, within the recognized 1691 proteins, 630 were involved in protein interactions, and these interacting proteins were further divided into six clusters (Fig 13A and S8 Table).

It was noteworthy that the sequences of 116 proteins associated with lipid metabolism pathway were subjected to further analysis using the STRING database (Fig 13B and S8 Table); of those, 38 interacted proteins were grouped into six subclusters. Cluster 1 included 10 proteins related to fatty acid catabolic process, such as ACX1 (Peroxisomal acyl-coenzyme A oxidase 1-like, Unigene097289), PKT3 (3-Ketoacyl-CoA thiolase 2, Unigene060972/Unigene088371/Unigene088372), AIM1 (Enoyl-CoA hydratase, Unigene069968), ACAT2 (Acetyl-CoA C-acetyltransferase protein, Unigene059463), IBR3 (Acyl-CoA dehydrogenase, Unigene090984/Unigene090985), and ACS (Acetyl-coenzyme A synthetase, Unigene073223). Cluster 2 included seven proteins that participated in steroid biosynthesis pathway; these were classified into four partners, named AT5G13980 (Glycosyl hydrolase family 38 protein, Unigene042399/Unigene055833), AT3G26720 (Glycosyl hydrolase family 38 protein, Unigene082277), AT3G54440 (Glycoside hydrolase family 2 protein, Unigene098848), AT5G13520 (Leukotriene A-4 hydrolase homolog isoform X1, Unigene072987), ALDH2B4 (Aldehyde dehydrogenase family 2 member B4, Unigene087228), OPCL1 (4-Coumarate-CoA ligase-like 5, Unigene063954), and LACS7 (Long-chain acyl-CoA synthetase 1, Unigene073239/Unigene074353). AT3G54440 interacted with AT5G13980, AT3G26720, and AT5G13520. Cluster 3 included 5 proteins involved in glycerophospholipid metabolism, for instance, POP2 (Pyridoxal phosphate (PLP)-dependent transferases superfamily protein, Unigene055866), ALDH5F1 (Succinate-semialdehyde dehydrogenase, Unigene060001), GLYR1 (Glyoxylate/succinic semialdehyde reductase 1, Unigene054958), ICL (Isocitrate lyase, Unigene072543), and MLS (Malate synthase, Unigene095897). Moreover, four lipid transport associated-proteins were defined to cluster 4, HMG1 (3-Hydroxy-3-methylglutaryl coenzyme A reductase, Unigene071777), IPP1 (Isopentenyl diphosphate isomerase, Unigene079546), HDR (Hydroxymethylbutenyl diphosphate reductase, Unigene055751), and AT3G54250 (Diphosphomevalonate decarboxylase MVD2-like, Unigene061298). Cluster 5 consisted of four proteins catalyzing the condensation reaction of fatty acid synthesis, FAB1 (Ketoacyl-ACP synthase II, Unigene099893), FaTA (Acyl-ACP thioesterase, Unigene065077), MOD1(Enoyl-[acyl-carrier-protein] reductase [NADH], Unigene068643), and EMB3147 (Malonyl-CoA:ACP transacylase, Unigene051391). Cluster 6 also contained two proteins associated with fatty acid activation, AT4G05160 (4-Coumarate-CoA ligase-like 7, Unigene060784) and ALDH2C4 (Aldehyde dehydrogenase, Unigene065635).

### Expression detection by qRT-PCR analysis

**Fig 14:**
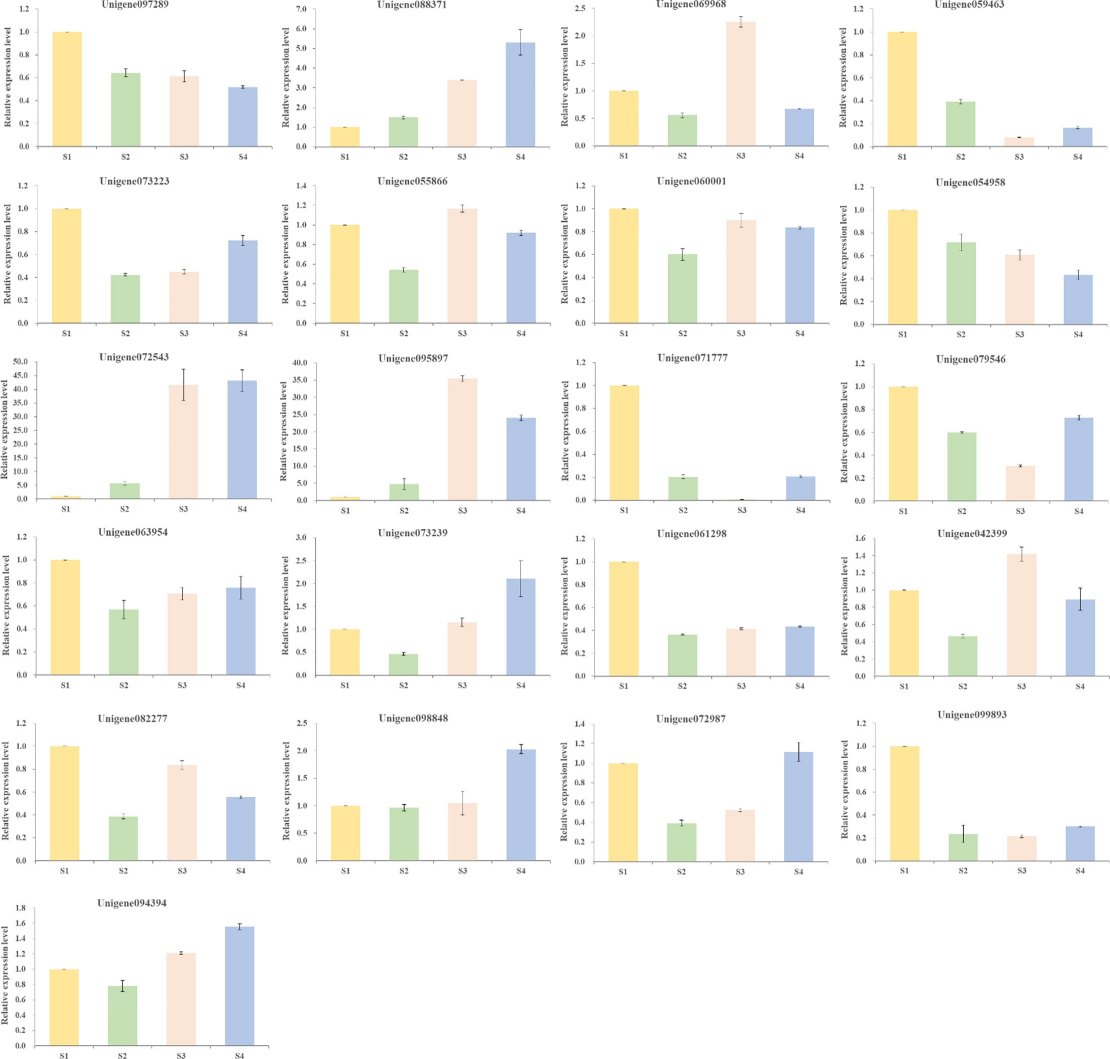
qRT-PCR quantification of the relative expression of unigenes encoding target proteins from *C*. *oleifera* seeds at four different developmental stages. To determine relative fold differences for the unigenes involved in lipid metabolism, their Ct values were normalized to the Ct value for Actin gene (GAPDH). The gene expression levels of S1 were set to 1, and relative expression levels were calculated relative to a calibrator using the formula 2^-ΔΔCt^. The detailed results for these unigenes are listed in S10 Table.

To further explore the correspondence between a protein and its mRNA expression pattern, and to identify the key candidate proteins related to lipid metabolism during *C*. *oleifera* ripening, 21 representative unigenes encoding target proteins were selected based on proteomic data to perform qRT-PCR at four different developmental periods. The results showed that the expression levels of these unigenes were diverse to different growth stages (Fig 14 and S10 Table). Among the differentially regulated proteins, the expression levels of Unigene088371 encoding PKT3 and Unigene072543 encoding ICL increased accompanying seed maturity, which were opposite to Unigene097289 and Unigene054958. Intriguingly, Unigene069968 encoding AIM1, Unigene055866 encoding POP2, Unigene095897 encoding MLS, and Unigene042399 encoding AT5G13980 reached their highest expression levels in S3, while being down-regulated slightly in S4. In the meantime, Unigene073239 encoding LACS7, Unigene098848 encoding AT3G54440, Unigene072987 encoding AT5G13520, and Unigene094394 encoding EMB1873 which might be conducive to fatty acid biosynthesis, were down-regulated in S2, whereas the same proteins were up-regulated in S4. It was worthy of note that the expression levels of Unigene072543 and Unigene095897 in S3 and S4 were distinctly higher than those in S1 and S2.

### Fatty acids profile in the *C*. *oleifera* oil

**Fig 15:**
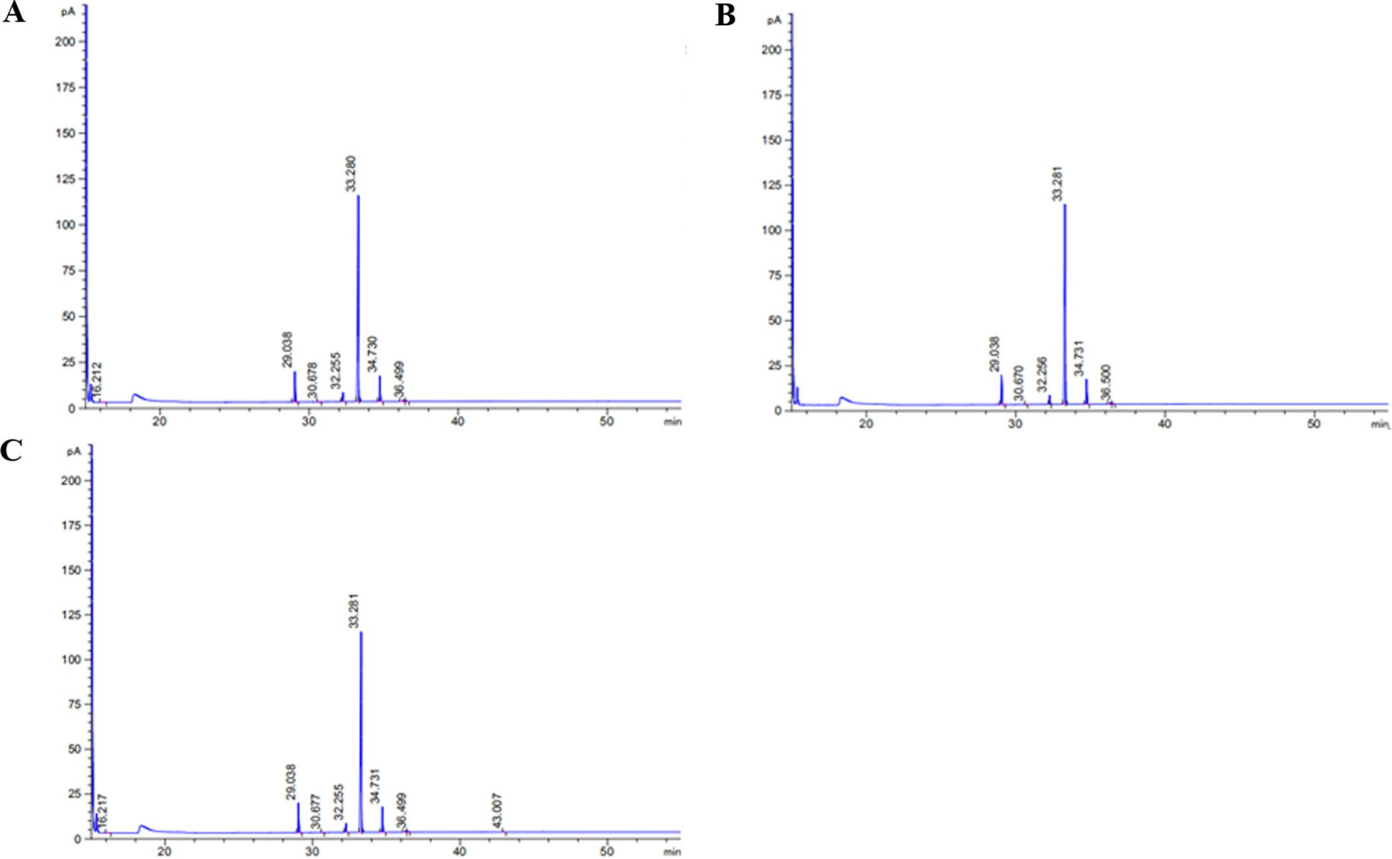
**Total ion chromatograms showing the fatty acids profiles of *C*. *oleifera* oils in three biological repetitions (A, B, and C).** The identification of the highest peak was oleic acid, followed by palmitic acid and α-linoleic acid.

To evaluate the quality of *C*. *oleifera* oil from Hainan Island, we extracted and measured the total oil content of seeds at maturity stage and detected the corresponding fatty acid compounds based on GC-MS analysis. The total ionization chromatogram of fatty acid methyl ester standards is shown in S8 Fig, and regression equations were calculated according to retention times and peak areas of standard substances (S11 Table). The typical fatty acid compositions of *C*. *oleifera* oils are listed in Table 2, and their total ion current chromatograms are presented in Fig 15. The results demonstrated that the oil content of dry seed was up to 42.01%. In addition, monounsaturated fatty acids (55.091 g/100g) constituted the main group of fatty acids present, followed by saturated fatty acids (9.862 g/100g) and polyunsaturated fatty acids (6.928 g/100g), in that order. The determined *C*. *oleifera* oil contained significant amounts (54.689 g/100g) of oleic acid (C18:1), similar to olive oil. Linoleic acid (C18:2) was also the one of dominant fatty acids, and its concentration was 6.662 g/100g. Among the polyunsaturated fatty acids, linolenic acid (0.266 g/100g) was also detected in *C*. *oleifera* oil from Hainan Island. Furthermore, palmitic acid and stearic acid played important roles in saturated fatty acids, and their concentrations were 7.905 g/100g and 1.931 g/100g, respectively. The correlations among different fatty acids of *C*. *oleifera* oils, are presented in Table 3. Stearic acid (C18:0) was significantly positively correlated with oleic acid (*p* < 0.01; r^2^ = 1.000**), but negatively correlated to eicosenic acid (C20:1) (*p* < 0.05; r^2^ = -0.998*). Also, negative correlations were found between linoleic acid and eicosenic acid (p < 0.05; r2 = -0.998*), as well as eicosenic acid and total fatty acids (*p* < 0.05; r^2^ = -0.997*).

**Table 2:** The content of fatty acid composition obtained for *C*. *oleifera* oils.

No.	Fatty acids	Absolute content (g/100g)
1	Palmitic acid (C16:0)	7.905 ± 0.022
2	Palmitoleic acid (C16:1)	0.026 ± 0.001
3	Margaric acid (C17:0)	0.025 ± 0.001
4	Stearic acid (C18:0)	1.931 ± 0.015
5	Oleic acid (C18:1)	54.689 ± 0.242
6	Linoleic acid (C18:2)	6.662 ± 0.016
7	α-Linolenic acid (C18:3)	0.266 ± 0.003
8	Eicosenic acid (C20:1)	0.360 ± 0.002
9	Tetracosenic acid (C24:1)	0.017 ± 0.001
	Saturated fatty acids	9.862 ± 0.024
	Monounsaturated fatty acids (MUFA)	55.091 ± 0.240
	Polyunsaturated fatty acids (PUFA)	6.928 ± 0.016
	Oleic acid/linoleic acid	8.209 ± 0.048
	MUFA/PUFA	7.952 ± 0.048

Note: Each value is expressed as the mean ± standard deviation.

**Table 3:** Correlation coefficients between different fatty acids of *C*. *oleifera* oils.

Fatty acids	C16:0	C16:1	C17:0	C18:0	C18:1	C18:2	C18:3	C20:1	C24:1	Total
C16:0	1									
C16:1	-0.723	1								
C17:0	0.909	-0.945	1							
C18:0	-0.213	-0.522	0.214	1						
C18:1	-0.209	-0.525	0.217	1.000**	1					
C18:2	0.980	-0.569	0.807	-0.404	-0.401	1				
C18:3	-0.366	0.908	-0.721	-0.831	-0.833	-0.172	1			
C20:1	0.148	0.577	-0.277	-0.998*	-0.998*	0.343	0.866	1		
C24:1	-0.996	0.655	-0.866	0.303	0.300	-0.994	0.277	-0.240	1	
Total	-0.076	-0.635	0.347	0.990	0.991	-0.274	-0.986	-0.997*	0.169	1

Note

** Correlation is significant at the 0.01 level.

* Correlation is significant at the 0.05 level.

## Discussion

### Annotation and network analyses of genes and proteins provided a global insight into the transcriptome and proteome of *C*. *oleifera*

Usually, the regulation processes from mRNA to protein are extraordinary complicated. Protein-mediated post-transcriptional and post-translational processes result in the final cellular products, compared to genome and transcriptome. However, the abundance level of protein species commonly depends on non-coding RNAs, mutation, degradation, protein interaction, post-translational modification, and DNA methylation [42,43]. Actually, a combined transcriptomic and proteomic analysis could indicate a reference catalog with great contiguity, wholeness, and accuracy for researchers and could provide an efficient way to understand how transcribed mRNA is manifested at the protein level [37].

In past decades, it was accompanied by the development of remarkable genomics, transcriptomics, and proteomics, research on *C*. *oleifera* as an important oilseed crop for both basic as well as application has been steadily increasing. Nevertheless, the availability of large-scale proteomic and transcriptomic data for mature seeds *C*. *oleifera* from Hainan Island has been very limited. Therefore, in the present study, protein identification relied on transcriptome database, and the quality of transcriptome assembly and sequencing played a key role in following analyses; the percentages of clean reads, Q30 values, and GC content were commonly used as important indicators to assess transcriptome quality [44]. A total of 59,391 transcripts were de novo assembled using Trinity software among three biological replicates, with 1025 bp of N50 value, 47.39% of GC percentage, and 94.53% of Q30 percentage. These results demonstrated that our transcriptome quality was high enough for further analysis. Subsequently, our improved protein extraction method was shown to be appropriate for the *C*. *oleifera* seeds [45].

Lately, several authors have investigated the edibility and quality of *C*. *oleifera* oil, indicating that the fatty acid composition of seeds was the most important factor to evaluate quality traits of the oil [46]. In addition, fatty acid metabolism processes have been reported to be pivotal during other natural products biosynthesis initiation, and they play crucial roles in translation, carbohydrate metabolism, abiotic stress responses, catalysis, and glycolysis [47]. Accordingly, studies on those genes/proteins related to lipid metabolism pathway of *C*. *oleifera* mature seeds from Hainan Island are extraordinarily important.

In this study, we conducted a primary integrated analysis of unigenes and proteins annotated by Nr, Swiss-Prot, KOG, KEGG databases, and the Blast2GO program. We found that 825, 999, 849, 523, and 868 members (unigenes/proteins) were co-expressed in the transcriptome and proteome, respectively (S9 Fig). There were 42 shared members that participated in lipid metabolism. In more detail, there were 691 unigenes involved in lipid transport and metabolism by KOG classification; however, only 42 unique proteins were expressed, such as acyl-coenzyme A thioesterase 9 (Unigene082845), malonyl-CoA: ACP transacylase (Unigene051391), oxalate-CoA ligase (Unigene091584), 4-coumarate-CoA ligase-like 5 (Unigene063954), phospholipase A-2-activating protein (Unigene093392), acetyl-CoA C-acetyltransferase protein (Unigene059463), and fatty acid beta-oxidation multifunctional protein (Unigene072566). The results indicated that there was a significant difference between levels of transcription and protein.

To further investigate the proteins associated with fatty acid biosynthesis, which could serve as a foundation to understand the mechanisms of fatty-acid metabolism in oil crops [48], KEGG pathway analysis was carried out. As shown in the results, a total of 522 unigenes were involved in lipid metabolism, whereas just 17 special unigenes were translated into proteins. It was remarkable that 31 unigenes were related to biosynthesis of unsaturated fatty acids at the transcriptional level, while only a few proteins in this pathway were found. In addition, there were 23 unigenes associated with linoleic acid metabolism, but merely three proteins (glycol-hydro-3 domain-containing protein (Unigene089382), nitrilase (Unigene058187), and 24-methylenesterol C-methyltransferase 2 (Unigene066313)) were identified from protein database. There were 55 unigenes participating in alpha-linolenic acid metabolism, but barely one protein (Uricase-2 isozyme, Unigene073019) was recognized, signifying that regulatory process from mRNA to protein also influenced the pathway of protein production.

There were 9528 expressed unigenes that involved in metabolic processes at the transcriptome level, with 326 specific proteins at the proteome level. Among these, 228 proteins took part in primary metabolic process, including 43 proteins associated with lipid metabolic process. From GO categories for the identified proteins related to regulation of lipid biosynthetic process, we also found several important proteins besides the ones mentioned above. For example, lipid catabolic process was regulated by cysteine protease RD19D (Unigene080281). Non-symbiotic hemoglobin 2 (Unigene044488) was involved in neutral lipid biosynthetic process. Late embryogenesis abundant protein D-34-like (Unigene060140) and actin-interacting protein 1-2-like (Unigene072617) played a part in the positive regulation of lipid biosynthetic process; conversely, biotin-lipoyl domain-containing protein (Unigene060190) participated in the negative regulation of lipid biosynthetic process. Remarkably, with regard to cellular components, the first group of identified proteins was located in the cell, followed by proteins in the cytoplasm and in organelles such as chloroplasts. Cytoplasm is the main area of life activities, and chloroplasts are the organelles of endosymbiotic origin that play an important role in plants [49]. The biosynthesis of saturated fatty acids occurred in the cytoplasm of plants, and this is catalyzed acyl-CoA carboxylase (ACCase) and fatty acid synthetase complex (FAS). The chloroplast is responsible for converting palmitic acid into stearic acid. These above two critical proteins were all identified in this study.

Protein-protein interaction analysis of 116 proteins involved in lipid metabolic process showed 38 specific proteins with strong relationships, among which PKT3, ACAT2, IBR3, and GLYR1 were the central proteins of the interaction network due to their interactions with many other proteins [50]. Furthermore, 21 special unigenes encoding lipid-associated proteins were selected to reveal the dynamic gene expression profiles of *C*. *oleifera* during different developmental periods by using qRT-PCR analysis. The expression levels of Unigene088371 and Unigene072543 increased significantly accompanying seed maturity, and Unigene073239, Unigene098848, Unigene072987, and Unigene094394 were down-regulated in S2, whereas they reached their highest expression levels in S4, indicating that these proteins might play important roles in fatty acid biosynthesis and degradation, and that they need to be further studied to identify new multienzyme complexes and to reveal corresponding regulatory mechanisms related to fatty acid metabolism.

### Most enzymes in *C*. *oleifera* are key proteins involved in fatty acid metabolism

The most prevalent compounds in *C*. *oleifera* oil were fatty acids, which affect the final quality of oils and fats as well as human health. It is known that high amounts of oleic acid are important due to lowering of cholesterol and triglycerides, and these effects are helpful in preventing cancer, cardiovascular diseases, and autoimmune disorders [51]. Linoleic acid, the second main unsaturated fatty acid, is usually thought to suppress immune responses, and it may have benefits for asthma and inflammation [52,53]. As expected, the content of these two unsaturated fatty acids of *C*. *oleifera* oil were relatively high, which was consistent with the result of Li et al. (2011) [54]. Likewise, linolenic acid was inversely related to blood pressure in a large cross-sectional study; its content detected by GC-MS complied with the international nutritional standards regarding “Omega meals” [54,55]. In addition, the ratio of monounsaturated fatty acids to polyunsaturated fatty acids (7.952), especially oleic acid to linoleic acid ratio (8.209), which also could be used as an important indicator of the oxidative stability in *C*. *oleifera* oil, was similar to that obtained in olive oil [56]. Intriguingly, there were significant correlations among some fatty acids. In view of this, the functional study of fatty acid metabolism proteins in oil crops played a crucial role in improving oil quality.

Fatty acid biosynthesis is an essential step, and many important proteins are associated with this pathway. Malonyl-CoA:ACP transacylase (MCAT), a mercaptosylase encoded by *mact* gene, is mainly responsible for transferring acyl chains from CoA thioesters to acyl carrier proteins (ACP), so this protein plays an important role in the biosynthesis of fatty acids as well as polyketide [57,58]. Acyl carrier protein (ACP) is a key protein in the control of the synthesis of long-chain saturated fatty acids via catalyzing palmitic acid to stearic acid or to very-long-chain saturated fatty acids, with the view to regulate the ratio of saturated fatty acid to unsaturated fatty acid [59]. By dehydrogenating stearic acid to form oleic acid, stearoyl-ACP desaturase (SAD) performs an essential function in the formation of unsaturated fatty acids [60], and hence directly determines the total content of unsaturated fatty acids [59].

Numerous proteins were engaged in the fatty acid decomposition by the β-oxidation cycle pathway. Acyl-CoA thioesterases (ACOTs) belong to a family of enzymes that catalyzes the hydrolysis of acyl-CoA to free fatty acid, providing the potential to regulate intracellular levels of fatty acids [61]. Furthermore, ACOTs are auxiliary enzymes in the oxidation of several lipids in peroxisomes [62]. Mitochondrial fatty acid oxidation is the major method to obtain energy from fatty acid metabolism. This procedure is significant to providing fatty acid-derived energy and acetyl-CoA for plant tissues, and its key step is the dehydrogenation of acyl-CoA ester by a chain length-specific enzyme, the acyl-CoA dehydrogenase (ACADs) [63]. The cellular activities are usually regulated by lipoxygenase (LOX), which is an enzyme crucial for linoleic acid metabolic in higher plants [64]. Moreover, this enzyme plays a role in the synthesis of oxylipins. Mikulska-Ruminska K found that there existed LOX specific to most of the available oxidizable positions on linoleic acid and arachidonic acid [65].

Significantly, fatty acids are important energy resources for organisms. If the oxidative pathway of fatty acid was obstructed, the body would not get enough energy [66]. The correlative research shows that enoyl-CoA hydratase (ECH) catalyzes the second step in the physiological β-oxidation pathway of fatty acid metabolism [67,68]. It is worth noting that the energy provided from fatty acids to organisms could be blocked without this enzyme, and the absence of this protein would lead to an increase in levels of unsaturated fatty acids, especially oleic acid and linoleic acid [69]. Aldehyde dehydrogenase (ALDH) is used as a reliable marker to separate stem cells and has been stated to play an important role in cell proliferation and protection [70,71]. Previous studies have indicated that ALDH belonging to the class of oxidoreductase could oxidize the aldehyde material to the corresponding carboxylic acid and could reduce the peroxidation response of aldehyde material [72]. In addition, Huang et al. (2019) also showed that this enzyme had a critical role in the degradation process of fatty acids [73].

In this work, the proteins mentioned above were all recognized in mature seeds of *C*. *oleifera* using a shotgun qualitative approach that was sensitive enough to detect scarce proteins [74]. Based on the presence of these proteins, *C*. *oleifera* oil should be considered as a health-promoting food oil with good nutritional properties. Nevertheless, the low ratio of oil production was still an important factor restricting the development of a *C*. *oleifera* industry in Hainan Island [75]. The lipid-associated proteins found in this research might be helpful to the synthetic regulation of its oil yield by genetic engineering techniques, resulting in potential application in the agriculture. In addition, some other proteins with unknown specific functions were also identified in this study, and these need to be explored in depth.

The expression levels of these proteins participated in fatty acid biosynthesis are affected by many factors, including plant varieties, climatic conditions, harvest times, picking methods, and oil-producing technologies. Yao et al. (2013) reported that the total content of linolenic acid and oleic acid increased with the decrease of latitude, while the content of linoleic acid decreased first and then increased with the decrease of latitude [76]. Luo et al. (2012) demonstrated that the oleic acid increased slowly in the initial stage of fat accumulation; moreover, compared with artificial harvesting, the oleic acid content was higher by the method of natural fruit-drop harvesting [77]. Zhang et al. (2013) found that the content of unsaturated fatty acids by cold pressing was the highest, followed by aqueous enzymatic extraction [78]. Therefore, comparative proteomics in different conditions could help fully exploit the specific proteins related to fatty acid biosynthesis and elaborate the complex and continuous synergistic changes of corresponding metabolic regulatory pathways, necessitating further study.

## Conclusions

Currently, increasing attention has been paid to the health and nutritional benefits of *C*. *oleifera* thanks to it being rich in fatty acids. This research was an attempt to systematically investigate the transcriptome and proteome of *C*. *oleifera* mature seeds from Hainan Island. A total of 59,391 transcripts, 40,500 unigenes, and 1691 protein species were identified using RNA-seq technology and shotgun proteomic method. Analyses of biological function annotations, correlations from transcriptome to proteome, and protein-protein interactions were also carried out. The results indicated that these specific proteins were divided into 24 groups based on the KOG functional classification, with the great majority being relevant to posttranslational modification, protein turnover and chaperones, carbohydrate transport, and metabolism. Then, the identified proteins were categorized by GO analysis, illustrating that single-organism, cellular, and metabolic processes were the relatively large categories. Furthermore, 823 proteins were mapped to 17 KEGG pathways, and the most abundant proteins were annotated to nucleotide metabolism pathway followed by translation and amino acid metabolism pathways. It was noteworthy that there were 116 proteins related to lipid metabolism, among which 38 specific proteins were involved in protein-protein interactions. The expressions of 21 candidate unigenes encoding target proteins were diverse to different growth stages from the qRT-PCR analysis. Overall, the conjoint analysis of transcriptome and proteome offered a complete picture with regard to the pathways of transport, metabolism, biogenesis, and posttranslational modification. Meanwhile, the study also deepened our understanding of the metabolic networks involved in fatty acid biosynthesis and degradation and paved the way to expedite *C*. *oleifera* breeding with higher oil content and better oil quality. Further studies should pay more attention to determine the detailed roles of lipid-associated proteins with unknown specific functions by using differential proteomics techniques.

## Supporting information

S1 Fig Expression pattern of genes from *C*. *oleifera* seeds, involved in the KEGG pathway of fatty acid metabolism (map 01212). Each circle indicates metabolites, with corresponding annotations on the side.
(TIF)

S2 Fig Expression pattern of genes from *C*. *oleifera* seeds, involved in the KEGG pathway of fatty acid biosynthesis (map 00061). The green and purple color in each column indicates identified and unidentified enzymes, respectively. The corresponding annotations are labeled on the side.
(TIFF)

S3 Fig Expression pattern of genes from *C*. *oleifera* seeds involved in the KEGG pathway of fatty acid degradation (map 00071). The green and purple color in each column indicates identified and unidentified enzymes, respectively. The corresponding annotations are labeled on the side.
(TIFF)

S4 Fig Expression pattern of genes from *C*. *oleifera* seeds involved in the KEGG pathway of alpha-linolenic acid metabolism (map 00592). The green and purple color in each column indicates identified and unidentified enzymes, respectively. The corresponding annotations are labeled on the side.
(TIFF)

S5 Fig Expression pattern of genes from *C*. *oleifera* seeds, involved in the KEGG pathway of biosynthesis of unsaturated fatty acids (map 01040). The green and purple color in each column indicates identified and unidentified enzymes, respectively. The corresponding annotations are labeled on the side.
(TIFF)

S6 Fig Expression pattern of genes from *C*. *oleifera* seeds, involved in the KEGG pathway of linoleic acid metabolism (map 00591). The green and purple color in each column indicates identified and unidentified enzymes, respectively. The corresponding annotations are labeled on the side.
(TIFF)

S7 Fig Expression pattern of genes from *C*. *oleifera* seeds, involved in the KEGG pathway of fatty acid elongation (map 00062). The green and purple color in each column indicates identified and unidentified enzymes, respectively. The corresponding annotations are labeled on the side.
(TIFF)

S8 Fig Total ionization chromatogram from the GC-MS analysis of fatty acid methyl ester standards. The numbers in this figure are as follows: 1, Methyl hexanoate (C6:0); 2, Methyl octanoate (C8:0); 3, Methyl decanoate (C10:0); 4, Methyl undecanoate (C11:0); 5, Methyl laurate (C12:0); 6, Methyl tridecanoate (C13:0); 7, Methyl myristate (C14:0); 8, Methyl myristoleate (C14:1n5); 9, Methyl pentadecanoate (C15:0); 10, Methyl pentadecenoate (C15:1n5); 11, Methyl palmitate (C16:0); 12, Methyl palmitoleate (C16:1n7); 13, Methyl heptadecanoater (C17:0); 14, Methyl heptadecenoate (C17:1n7); 15, Methyl stearate (C18:0); 16, Methyl elaidate (C18:1n9t); 17, Methyl oleate (C18:1n9c); 18, Methyl linolelaidate (C18:2n6t); 19, Methyl linoleate (C18:2n6c); 20, Methyl arachidate (C20:0); 21, Methyl γ-linolenate (C18:3n6); 22, Eicosenoic acid methyl ester (C20:1); 23, Methyl α-linolenate (C18:3n3); 24, Methyl heneicosanoate (C21:0); 25, Eicosadienoic acid methyl ester (C20:2); 26, Methyl behenate (C22:0); 27, cis-8,11,14-Eicosatrienoic acid methyl ester (C20:3n6); 28, Methyl erucate (C22:1n9); 29, cis-11,14,17-Eicosatrienoic acid methyl ester (C20:3n3); 30, Methyl arachidonate (C20:4n6); 31, Methyl tricosanoate (C23:0); 32, Docosadienoic acid methyl ester (C22:2n6); 33, Methyl tetracosanoate (C24:0); 34, Eicosapentaenoic acid methyl ester (C20:5n3); 35, Methyl tetracosenoate (C24:1n9); 36, Docosahexaenoic acid methyl ester (C22:6n3). Methyl butyrate (C4:0) was not detected.
(TIFF)

S9 Fig Conjoint analyses of unigenes (RNA-Seq) and proteins (iTRAQ) showing unique and shared members in the transcriptome and proteome. Venn diagram of the integrated analysis of unigenes and proteins annotated by Nr database, 825 members (unigenes/proteins) were co-expressed in the transcriptome and proteome (A); Venn diagram of unigenes and proteins annotated by the Swiss-Prot database, with 999 shared members (B); Venn diagram of unigenes and proteins annotated by the KOG database, with 849 shared members (C); Venn diagram of unigenes and proteins annotated by the Blast2GO program, with 868 shared members (D); Venn diagram of unigenes and proteins annotated by the KEGG database, with 523 shared members (E); Venn diagram of unigenes and proteins related to lipid metabolism, with 42 shared members (F).
(TIFF)

S1 Table The observation of important botanical traits of *C*. *oleifera* samples. Samples of *C*. *oleifera* were harvested during the 2018 season, including four different developmental periods of nutrition synthesis stage (S1, August 24th), fat accumulation stage (S2, September 24th), near mature stage (S3, October 24th), and full maturity stage (S4, November 24th).
(DOCX)

S2 Table The expression information of unigenes from *C*. *oleifera* seeds and KOG classification by three independent RNA-Seq transcriptome analyses. Sheet 1, The unigenes were obtained by TGICL in three biological replicates (A, B, and C); Sheet 2, Annotation information of expressed genes from the Swiss-Prot protein database; Sheet 3, Annotation information of expressed genes from the NR protein database; Sheet 4, KOG classification for expressed genes; Sheet 5, Species distribution for expressed genes.
(XLSX)

S3 Table GO terms of the expressed genes from *C*. *oleifera* seeds. (XLSX)

S4 Table KEGG enrichment pathways of the expressed genes from *C*. *oleifera* seeds. (XLSX)

S5 Table The identification information of proteins from *C*. *oleifera* seeds and KOG classification by three independent shotgun proteome analysis. Sheets 1–3, The MS/MS identification information and amino acid sequences of proteins detected in three biological replicates (A, B, and C); Sheet 4, The MS/MS identification information and amino acid sequences of 1691 proteins detected three times; Sheet 5, KOG classification for identified proteins; Sheet 6, Species distribution for identified proteins; Sheet 7, Subcellular location for identified proteins.
(XLSX)

S6 Table GO terms of the identified proteins from *C*. *oleifera* seeds. (XLSX)

S7 Table KEGG enrichment pathways of the identified proteins from *C*. *oleifera* seeds. (XLSX)

S8 Table Protein-protein interactions of the identified proteins from *C*. *oleifera* seeds. According to STRING network analysis, 1182 of 1691 identified proteins were transformed into *Arabidopsis thaliana* proteins based on protein sequences; 630 proteins were involved in protein interactions, with a high confidence view (score 0.90). Accordingly, 100 of 116 lipid-related proteins were converted into *Arabidopsis thaliana* proteins, among which 38 proteins were mutually interactive, with the same confidence view.
(XLSX)

S9 Table Sequences of primers used to amplify genes from *C*. *oleifera* seeds involved in lipid metabolism pathway. A commonly used reference gene (GAPDH), was used to normalize the expression levels of target genes.
(XLSX)

S10 Table Relative expression information of unigenes encoding target proteins from *C*. *oleifera* seeds at four different developmental stages by qRT-PCR assay. To determine relative fold differences for the unigenes involved in lipid metabolism, their Ct values were normalized to the Ct value for the Actin gene (GAPDH). The gene expression levels of S1 were set to 1, and relative expression levels were calculated relative to a calibrator using the formula 2^-ΔΔCt^.
(XLSX)

S11 Table Linear regression data of fatty acid methyl ester standards. Regression equations ^a^: y = peak area of fatty acid methyl ester, x = concentration of fatty acid methyl ester, mg/l.
(XLSX)

S1 File The protein database of *C*. *oleifera* seeds from Hainan Island. The protein database was established by TransDecoder software, based on transcriptome data.
(FASTA)
