# Supplementary figures and images for: Complementary transcriptome and proteome profiling in the mature seeds of Camellia oleifera from Hainan Island
Source: PLoS One. 2020 Feb 6;15(2):e0226888. doi: 10.1371/journal.pone.0226888 (PMC7004384; doi:10.1371/journal.pone.0226888)

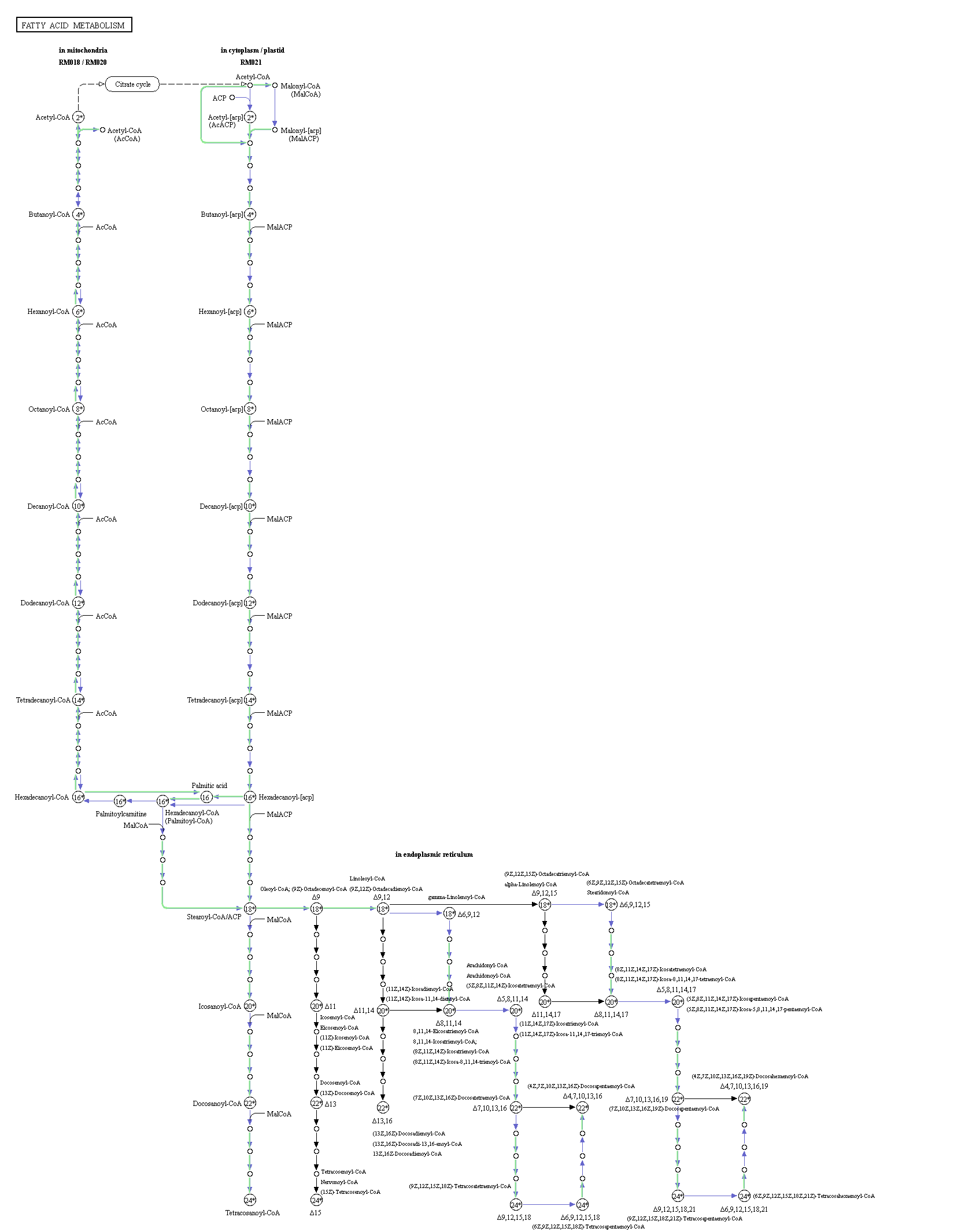

Supplement: S1 Fig — Each circle indicates metabolites, with corresponding annotations on the side. (TIF) [file pone.0226888.s001.tif]

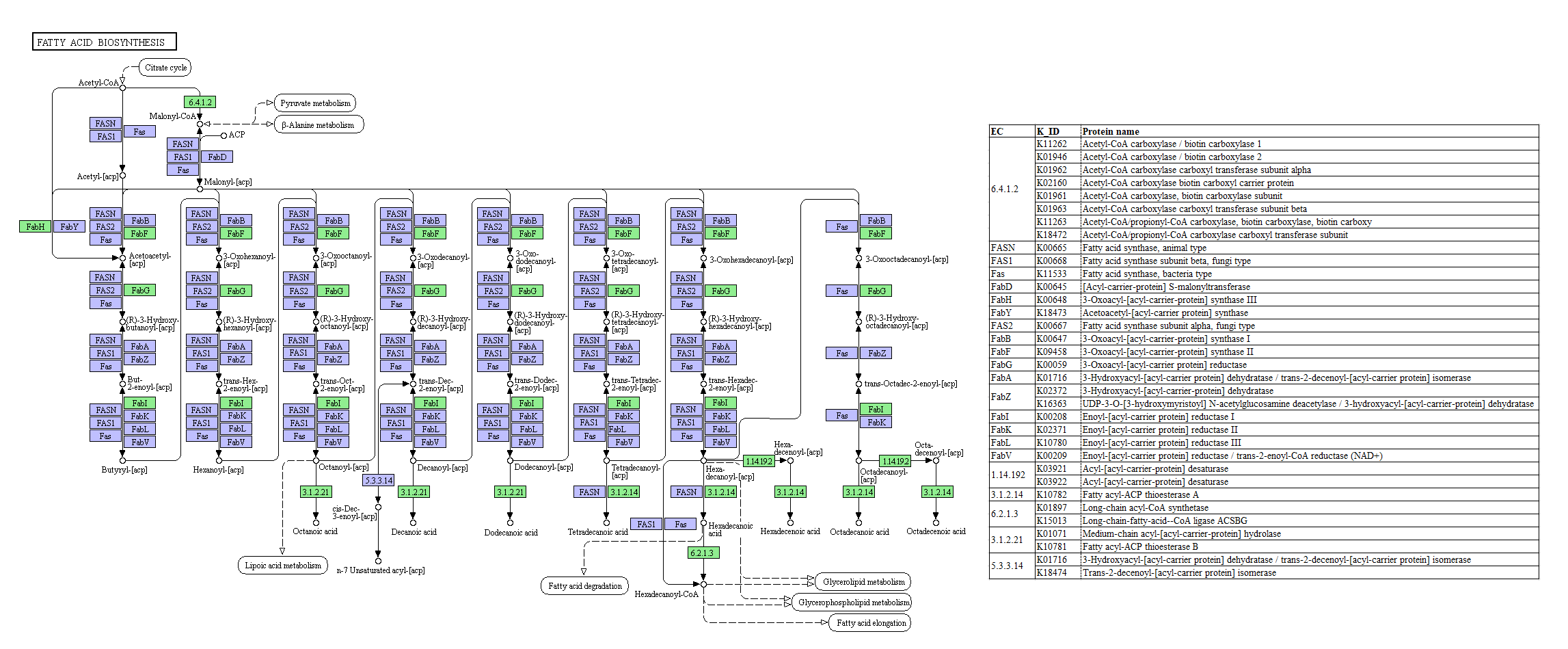

Supplement: S2 Fig — The green and purple color in each column indicates identified and unidentified enzymes, respectively. The corresponding annotations are labeled on the side. (TIFF) [file pone.0226888.s002.tiff]

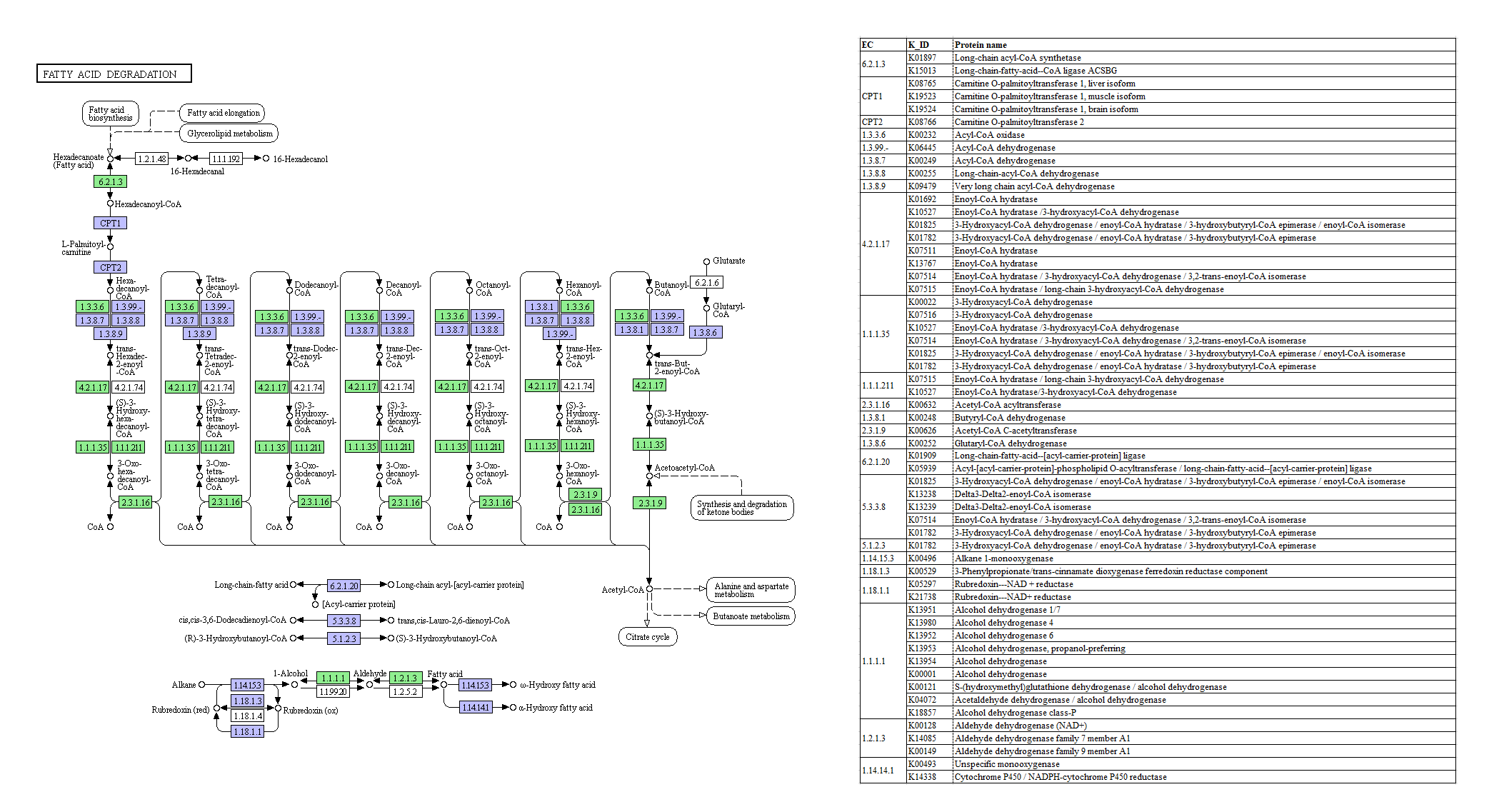

Supplement: S3 Fig — The green and purple color in each column indicates identified and unidentified enzymes, respectively. The corresponding annotations are labeled on the side. (TIFF) [file pone.0226888.s003.tiff]

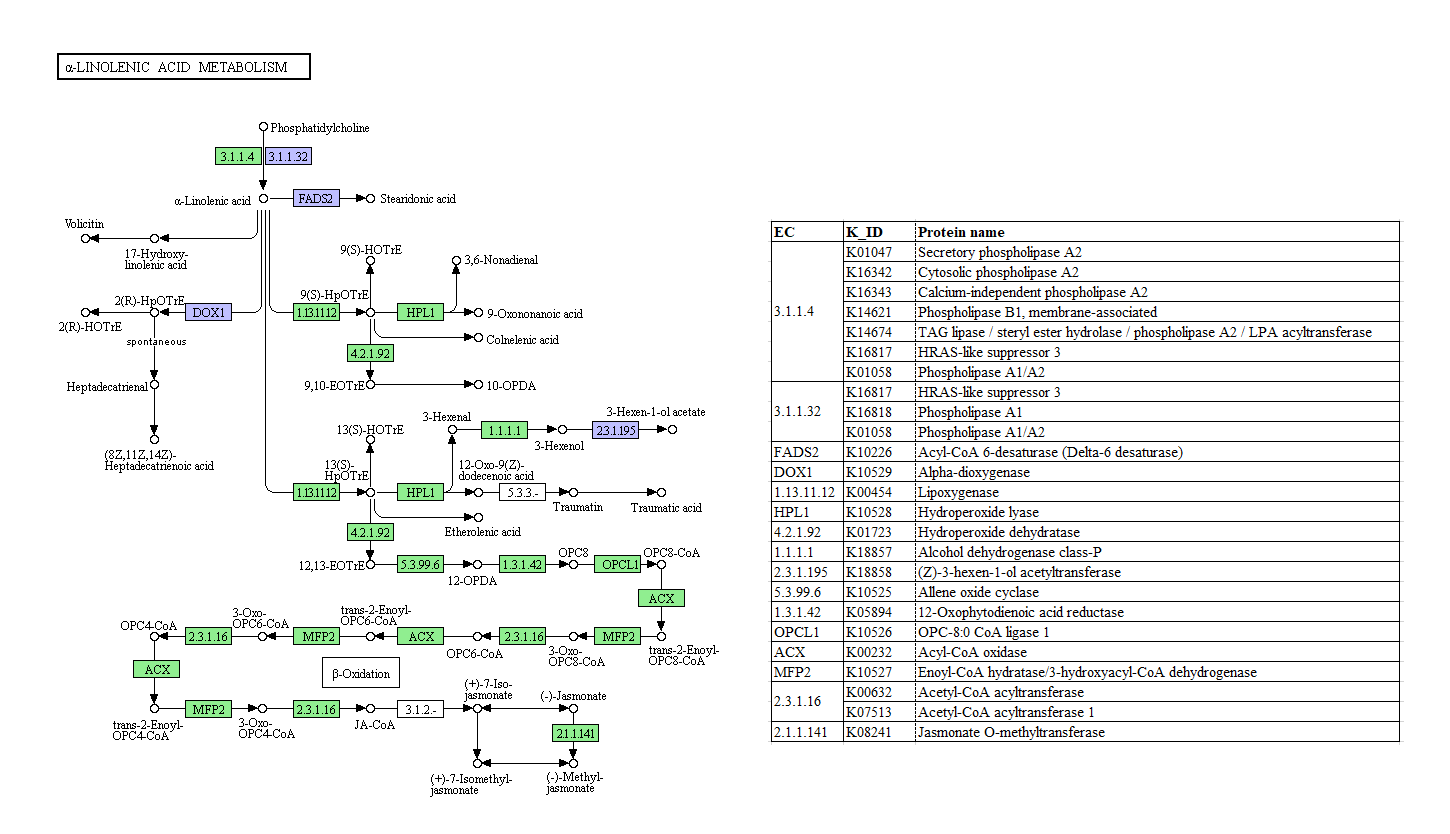

Supplement: S4 Fig — The green and purple color in each column indicates identified and unidentified enzymes, respectively. The corresponding annotations are labeled on the side. (TIFF) [file pone.0226888.s004.tiff]

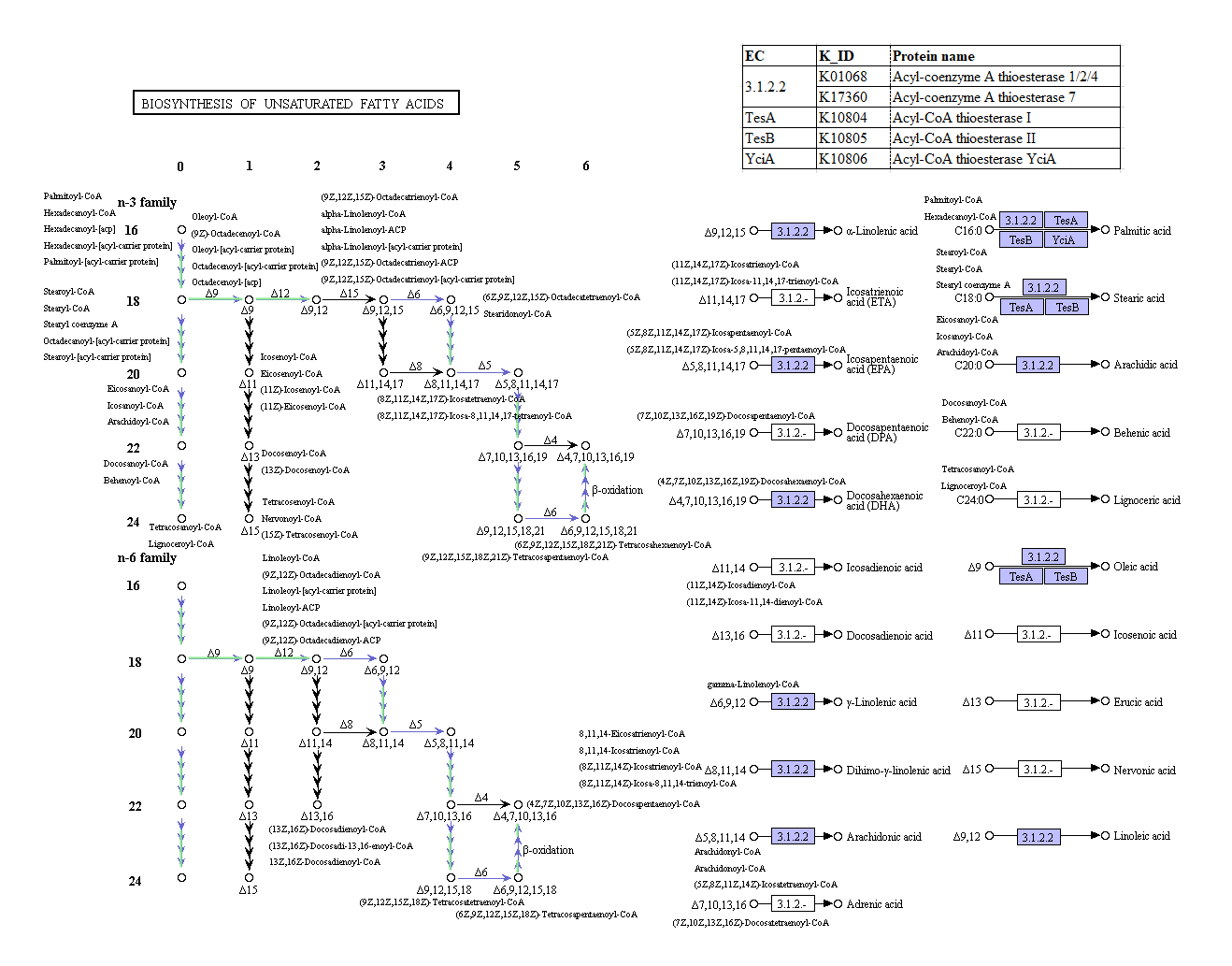

Supplement: S5 Fig — The green and purple color in each column indicates identified and unidentified enzymes, respectively. The corresponding annotations are labeled on the side. (TIFF) [file pone.0226888.s005.tiff]

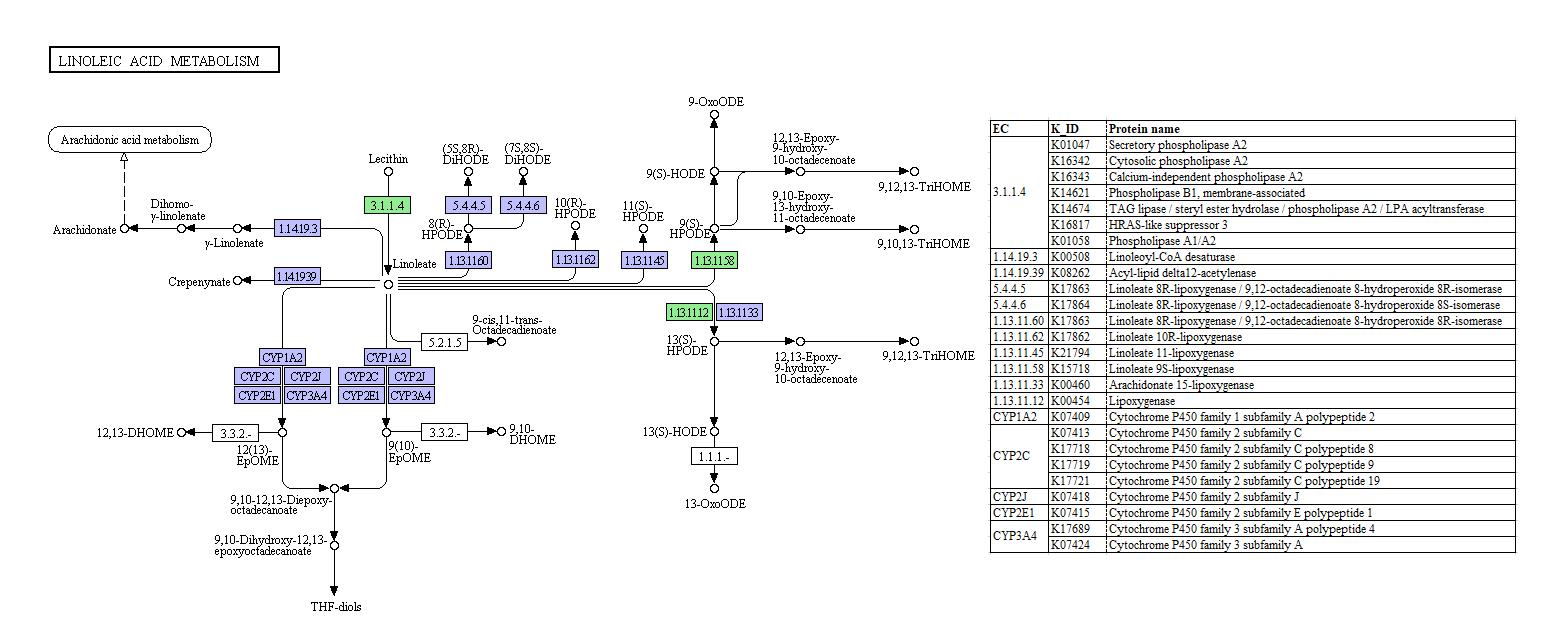

Supplement: S6 Fig — The green and purple color in each column indicates identified and unidentified enzymes, respectively. The corresponding annotations are labeled on the side. (TIFF) [file pone.0226888.s006.tiff]

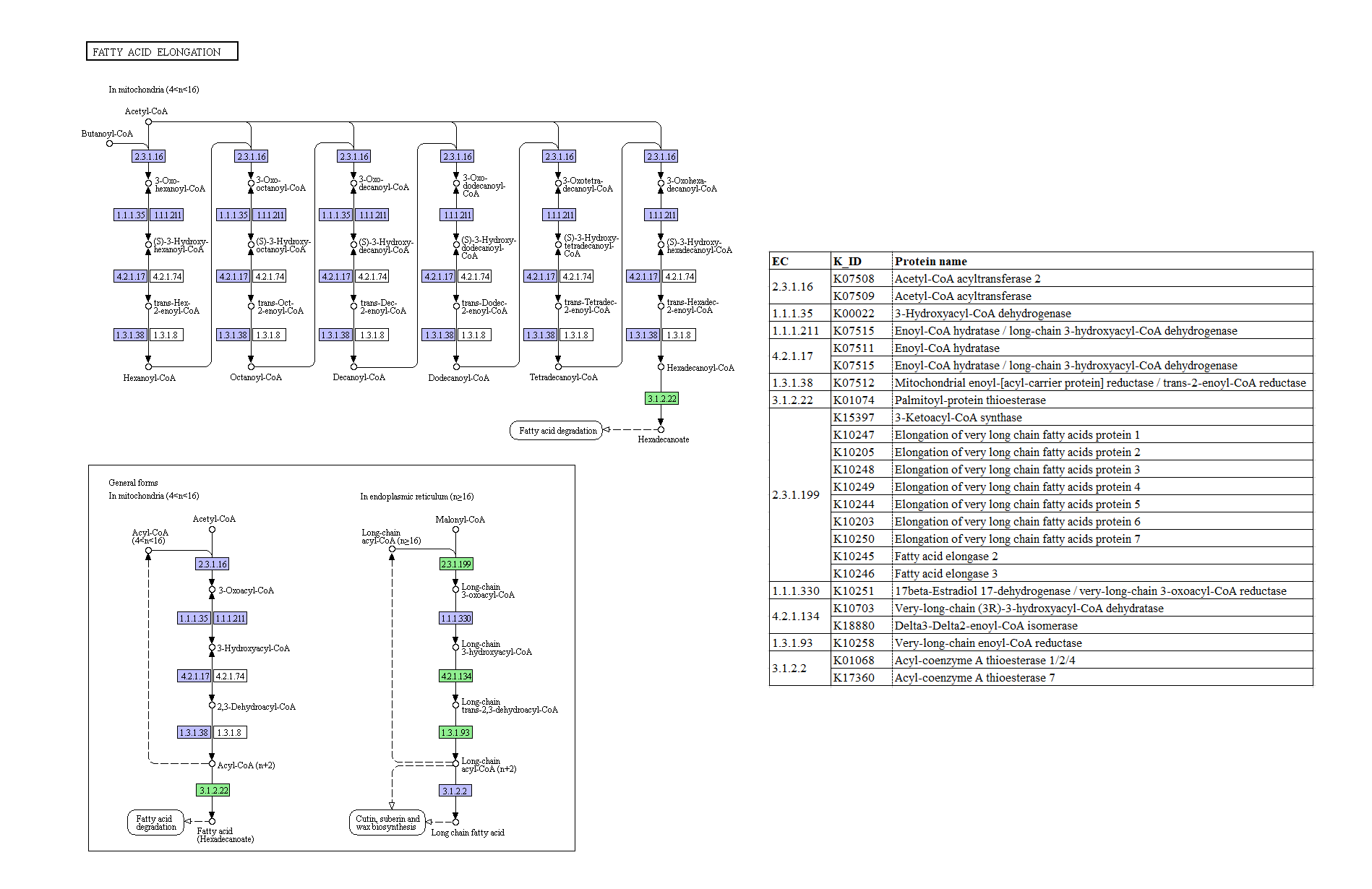

Supplement: S7 Fig — The green and purple color in each column indicates identified and unidentified enzymes, respectively. The corresponding annotations are labeled on the side. (TIFF) [file pone.0226888.s007.tiff]

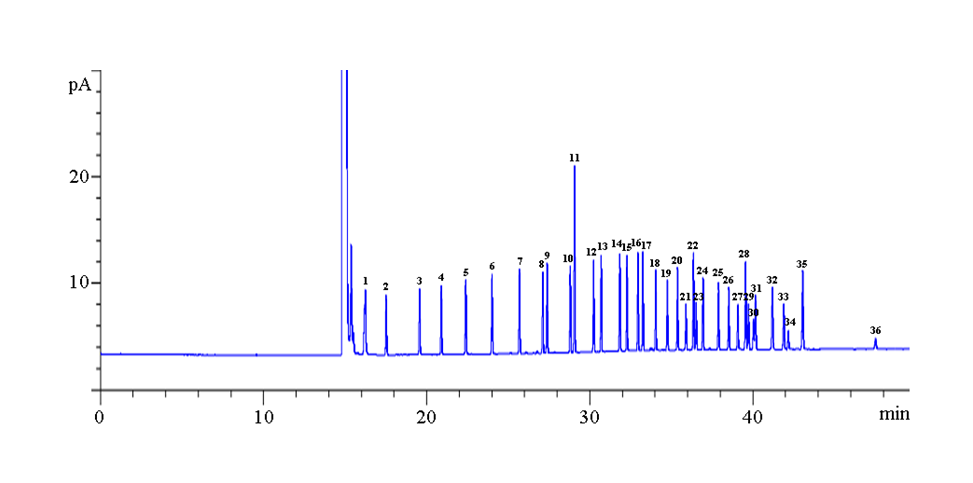

Supplement: S8 Fig — The numbers in this figure are as follows: 1, Methyl hexanoate (C6:0); 2, Methyl octanoate (C8:0); 3, Methyl decanoate (C10:0); 4, Methyl undecanoate (C11:0); 5, Methyl laurate (C12:0); 6, Methyl tridecanoate (C13:0); 7, Methyl myristate (C14:0); 8, Methyl myristoleate (C14:1n5); 9, Methyl pentadecanoate (C15:0); 10, Methyl pentadecenoate (C15:1n5); 11, Methyl palmitate (C16:0); 12, Methyl palmitoleate (C16:1n7); 13, Methyl heptadecanoater (C17:0); 14, Methyl heptadecenoate (C17:1n7); 15, Methyl stearate (C18:0); 16, Methyl elaidate (C18:1n9t); 17, Methyl oleate (C18:1n9c); 18, Methyl linolelaidate (C18:2n6t); 19, Methyl linoleate (C18:2n6c); 20, Methyl arachidate (C20:0); 21, Methyl γ-linolenate (C18:3n6); 22, Eicosenoic acid methyl ester (C20:1); 23, Methyl α-linolenate (C18:3n3); 24, Methyl heneicosanoate (C21:0); 25, Eicosadienoic acid methyl ester (C20:2); 26, Methyl behenate (C22:0); 27, cis-8,11,14-Eicosatrienoic acid methyl ester (C20:3n6); 28, Methyl erucate (C22:1n9); 29, cis-11,14,17-Eicosatrienoic acid methyl ester (C20:3n3); 30, Methyl arachidonate (C20:4n6); 31, Methyl tricosanoate (C23:0); 32, Docosadienoic acid methyl ester (C22:2n6); 33, Methyl tetracosanoate (C24:0); 34, Eicosapentaenoic acid methyl ester (C20:5n3); 35, Methyl tetracosenoate (C24:1n9); 36, Docosahexaenoic acid methyl ester (C22:6n3). Methyl butyrate (C4:0) was not detected. (TIFF) [file pone.0226888.s008.tiff]

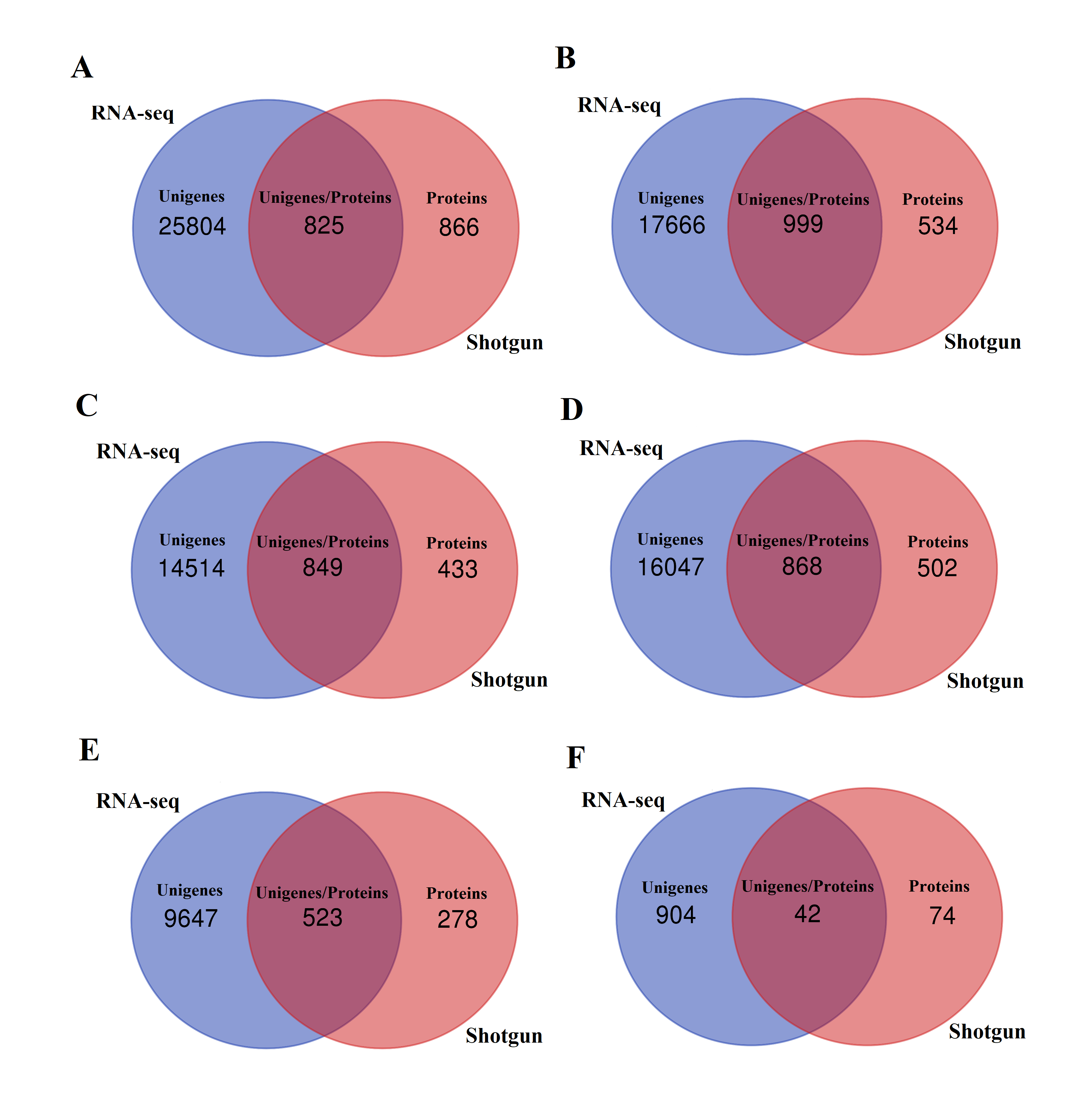

Supplement: S9 Fig — Venn diagram of the integrated analysis of unigenes and proteins annotated by Nr database, 825 members (unigenes/proteins) were co-expressed in the transcriptome and proteome (A); Venn diagram of unigenes and proteins annotated by the Swiss-Prot database, with 999 shared members (B); Venn diagram of unigenes and proteins annotated by the KOG database, with 849 shared members (C); Venn diagram of unigenes and proteins annotated by the Blast2GO program, with 868 shared members (D); Venn diagram of unigenes and proteins annotated by the KEGG database, with 523 shared members (E); Venn diagram of unigenes and proteins related to lipid metabolism, with 42 shared members (F). (TIFF) [file pone.0226888.s009.tiff]
