# Supplementary material for: Complementary transcriptome and proteome profiling in the mature seeds of Camellia oleifera from Hainan Island
Source: PLoS One. 2020 Feb 6;15(2):e0226888. doi: 10.1371/journal.pone.0226888 (PMC7004384; doi:10.1371/journal.pone.0226888)
Supplement: S1 Table — Samples of C. oleifera were harvested during the 2018 season, including four different developmental periods of nutrition synthesis stage (S1, August 24th), fat accumulation stage (S2, September 24th), near mature stage (S3, October 24th), and full maturity stage (S4, November 24th). (DOCX) [file pone.0226888.s010.docx]

| **S1 Table. The observation of important botanical traits of *C. oleifera* samples.** | | | | | | | | | | |
| --- | --- | --- | --- | --- | --- | --- | --- | --- | --- | --- |
| **No.** | **Sample** | **Fresh fruit weight (g)** | **Seed percentage in fresh fruit (%)** | **Oil content ratio in dry seed (%)** | **Fruit length (cm)** | **Fruit width (cm)** | **Fruit shape index** | | **Pericarp thickness (cm)** | |
| 1 | S1 | 34.183 | 17.074 | 25.523 | 3.402 | 4.077 | | 0.834 | | 0.627 |
| 2 | S2 | 61.556 | 25.318 | 28.971 | 4.411 | 5.023 | | 0.878 | | 0.636 |
| 3 | S3 | 79.312 | 19.130 | 41.244 | 4.511 | 5.562 | | 0.811 | | 0.655 |
| 4 | S4 | 88.185 | 21.860 | 42.012 | 4.749 | 5.750 | | 0.826 | | 0.761 |
